# Research Progress in Biomedical Materials

**DOI:** 10.3390/biom16060844

**Published:** 2026-06-09

**Authors:** Yuting Wang, Dianpeng Wang, Xinyue Ma, Yuqing Cui, Jing Liu, Wenyuan Fang

**Affiliations:** 1 School of Pharmacy, Jining Medical University, Rizhao 276800, China; wangyuting@stu.mail.jnmc.edu.cn (Y.W.); maxinyue@stu.mail.jnmc.edu.cn (X.M.); cuiyuqing@stu.mail.jnmc.edu.cn (Y.C.); 2 Lanshan Branch of Rizhao Ecological Environment Bureau, Rizhao 276807, China; wdianpeng@126.com

**Keywords:** biomedical materials, surface modification, clinical applications, drug delivery, tissue repair

## Abstract

Biomedical materials, which are engineered to interact safely and effectively with biological systems, serve as the foundation of modern medicine. They facilitate precise diagnostics, targeted therapies, tissue regeneration, and the functional restoration of damaged organs and tissues. Propelled by advancements in materials science, nanotechnology, and clinical understanding, this field is rapidly evolving from passive implants to intelligent, responsive, and bioactive systems. This review summarize recent breakthroughs in four crucial domains: hard-tissue repair, dynamic wound healing, spatiotemporally controlled drug delivery, and advanced surface engineering. This article rigorously assesses the persistent translational barriers, particularly the disparity between in vitro biocompatibility assays and clinical performance, the scalability constraints in manufacturing, and the fragmentation in regulatory frameworks and international standards. By connecting fundamental innovation with real-world clinical needs, this review functions as both a strategic reference for researchers and a practical resource for clinicians exploring the next generation of biomedical materials.

## 1. Introduction

Biomedical materials are specifically engineered substances that can interact in a safe and effective manner with biological systems for the purposes of disease diagnosis, therapy delivery, regeneration of damaged tissues or organs, restoration of lost functions, and enhancement of physiological performance. Their development lies at the cross-section of medicine, materials science, and engineering, which drives and accelerates clinical innovation. In the early stages, applications were dependent on basic natural materials such as wood, bone, and metals, which provided only limited biocompatibility and functionality. The field emerged as a distinct scientific discipline during the mid-20th century, spurred by breakthroughs including synthetic polymers, stainless-steel implants, and subsequently, bioactive ceramics and smart hydrogels. In 2020, the global biomedical materials market reached a value of $110 billion and is forecasted to surpass $200 billion by 2024, with a compound annual growth rate (CAGR) of 15.2%. Although North America accounts for the largest share, the Asia–Pacific region, led by the rapid growth in China and India, is currently the fastest-growing market, propelled by increasing healthcare investment, aging populations, and the advancement of domestic research and development capabilities.

Biomedical materials serve as the cornerstone of modern medicine, driving breakthroughs in diagnosis, treatment, rehabilitation, and prevention. Ranging from life-saving heart stents and infection-resistant orthopedic implants to bioactive dental prostheses and patient-matched craniofacial scaffolds, these materials not only replace damaged tissues but also restore physiological function, accelerate the healing process, and reestablish patients’ confidence and autonomy. In the management of chronic diseases, smart biomaterials facilitate precise and sustained drug delivery, such as glucose-responsive insulin depots, which revolutionize the daily care of millions of diabetes patients. Beyond mere replacement, they foster next-generation therapies, including vascularized tissue constructs grown from a patient’s own cells, 3D-bioprinted cartilage customized to anatomical contours, and nanocarriers that target tumors while safeguarding healthy tissue. These innovations not only enhance clinical outcomes but also reduce surgical invasiveness, shorten hospital stays, lower long-term healthcare costs, and stimulate high-value employment in biomanufacturing, regulatory science, and digital health. Ultimately, biomedical materials are not passive instruments but active facilitators of human resilience, promoting both individual well-being and population-level health equity.

As the global population ages, biomedical materials are assuming an increasingly crucial role. They are not only instrumental in treating age-related diseases and maintaining the health of the elderly but also in relieving the systemic burdens on healthcare systems. For instance, advanced artificial joints and orthopedic implants have restored mobility for millions of senior citizens, substantially enhancing their quality of life and mitigating chronic pain and disability. Beyond their clinical influence, these materials serve as catalysts for innovation in medical technology, expedite regulatory and manufacturing progress, and stimulate economic development in the healthcare sectors. With the rapid advancement in materials science, AI-driven design, and personalized medicine, the biomedical materials industry is on the verge of becoming a cornerstone of the next-generation health revolution, driving safer, smarter, and more equitable care for aging societies globally.

Biomedical materials can be traced back to approximately 3500 BC, when the ancient Egyptians utilized cotton fibers and horsehair as sutures, which are among the earliest known instances of purposefully applied biomaterials. By the end of the 19th century, advancements in antisepsis facilitated the clinical application of metals such as stainless steel for orthopedic implants. In the middle of the 20th century, a paradigm shift occurred with the introduction of synthetic polymers, including polyethylene and silicone rubber, which marked the beginning of the modern era of biomedical materials and enabled breakthroughs in artificial organs and implantable devices. In the late 20th and early 21st centuries, the increasing emphasis on biocompatibility and controlled biodegradability spurred innovations such as absorbable sutures and stimuli-responsive drug delivery systems. These advancements synergized with emerging digital tools, although they were not driven by AI itself. More recently, nanotechnology and 3D bioprinting have enhanced design precision and functional customization, thereby accelerating the development of patient-specific implants and tissue-engineered constructs. Looking forward, the interdisciplinary convergence across materials science, biology, data analytics, and engineering has the potential to bring about transformative changes in next-generation diagnostics, regenerative therapies, and personalized medicine [1].

Medical molecular materials are required to meet extremely strict performance criteria to guarantee safety, efficacy, and reliability in clinical applications. These requirements can be classified into five interrelated categories: (1) Biocompatibility and Safety: Materials should be non-toxic, non-carcinogenic, non-allergenic, and non-inflammatory. They should not trigger adverse immune responses or cause rejection. (2) Mechanical and Chemical Stability: Materials need to display mechanical properties that are customized to their intended functions. For example, artificial joint components should have high wear resistance and compressive strength, and they should maintain structural and functional integrity over time in vivo. For instance, coronary stents demand long-term radial strength and corrosion resistance in the dynamic vascular environment. (3) Processability: Materials must be suitable for advanced fabrication techniques, including precision machining, injection molding, and additive manufacturing, which can enable the reproducible production of complex, patient-specific devices and implants. (4) Sterilization Robustness: Materials must preserve their physical, chemical, and biological properties after standard sterilization methods (such as autoclaving, ethylene oxide gas, or gamma irradiation), without degradation, leaching, or surface alteration that might affect performance or safety. (5) Functionally Tuned Degradation (where applicable): For temporary implants, such as absorbable sutures, drug-eluting matrices, or scaffolds for tissue regeneration, the degradation kinetics must be precisely controllable. The degradation products should be non-toxic, metabolically benign, and efficiently cleared by physiological pathways [2].

Moreover, the production and utilization of biomedical materials must adhere to national and international regulatory standards, including ISO 10993-1 [3] for biocompatibility assessment and FDA requirements for medical devices. Every phase, ranging from raw material procurement and production to sterilization, labeling, and clinical application, must be meticulously documented to guarantee complete traceability and facilitate prompt root-cause analysis in the event that safety or performance issues occur. By fulfilling these strict criteria, biomedical materials safeguard patient safety, clinical effectiveness, and functional dependability, ultimately contributing to the provision of high-quality, reliable healthcare.

## 2. Major Categories of Biomedical Materials and Advancements in Their Applications

### 2.1. Medical Metallic Materials: Core Carriers for Mechanical Support

Medical metallic materials refer to metals and alloys that are specifically designed for utilization in healthcare applications. They are chosen due to their outstanding mechanical strength, biocompatibility, and resistance to corrosion within physiological environments. These materials assume crucial roles in orthopedic and dental implants, cardiovascular stents, surgical instruments, and temporary fixation devices, thereby facilitating structural repair, functional restoration, and tissue integration. As fundamental components of advanced medical devices and next-generation biohybrid organs, they integrate engineering precision with biological performance. Commonly used examples encompass austenitic stainless steels (e.g., 316L), commercially pure titanium and Ti-6Al-4V alloy, cobalt–chromium–molybdenum alloys, nickel–titanium shape-memory alloys, biodegradable magnesium alloys, as well as high-purity tantalum and zirconium.

Medical metal materials are of great significance not only for saving and enhancing lives but also for promoting healthcare innovation and socioeconomic development. In the field of orthopedics, stainless steel, which is highly regarded for its corrosion resistance, strength, and cost-effectiveness, has long been a reliable material for fracture fixation devices and surgical instruments. Titanium and its alloys possess superior biocompatibility, low density, corrosion resistance, and an elastic modulus similar to that of natural bone, thereby establishing them as the preferred choice for artificial joints, dental implants, and cardiovascular stents. However, their complex machining requirements lead to relatively higher costs. Cobalt–chromium alloys offer exceptional wear resistance and fatigue strength, making them suitable for load-bearing joint replacements; nickel–titanium shape-memory alloys facilitate minimally invasive device deployment (e.g., self-expanding stents); and biodegradable magnesium alloys hold promise for temporary implants such as bone fixation screws, although their clinical application is still in progress.

#### 2.1.1. Stainless Steel Material

This material is an iron-based stainless steel alloy, which is primarily strengthened by chromium, nickel, and molybdenum. These alloying elements act synergistically to enhance its mechanical strength, thermal stability, and resistance to deformation during both cold and hot working, thereby enabling cost-effective and high-precision manufacturing. Although it exhibits excellent formability and structural robustness for medical devices, its corrosion resistance and in vivo biocompatibility are still inferior to those of titanium and cobalt–chromium alloys. Clinically, it is extensively used in orthopedic implants, including bone plates, intramedullary nails, and fracture fixation systems, as well as reusable surgical instruments. For instance, Datti et al. have demonstrated via clinical research that stainless steel mesh is an effective material for repairing cranial defects and causes few adverse reactions [4].

In China, research efforts are concentrated on high-nitrogen, nickel-free stainless steels to eliminate nickel-induced sensitization and allergic responses. Simultaneously, surface modification techniques such as gas-phase and plasma nitriding are employed to enhance wear resistance and localized corrosion protection. For example, the stainless-steel material with a copper coating developed by Tripathi et al. can significantly reduce the infection rate of Gram-negative Escherichia coli and 99% of Gram-positive *Staphylococcus epidermidis*. This confirms that the performance of stainless-steel materials can be effectively enhanced through surface engineering methods, which can effectively prevent bacterial infections caused by surface contamination without leading to antibiotic resistance [5].

Internationally, high-entropy stainless steels, engineered with five or more principal metallic elements in near-equiatomic proportions, represent a state-of-the-art advancement. They offer an exceptional balance of strength and ductility, superior pitting resistance, and improved passivation stability. Several such alloys have now entered the clinical trial phases and are being scaled up for industrial translation [6]. Moreover, certain scholars have verified that silver-doped high-entropy nitride coatings are capable of enhancing the hydrophobicity of medical stainless steel, as well as its resistance against Pseudomonas aeruginosa and the novel coronavirus. However, they exhibit no inhibitory effect on Staphylococcus aureus. This experiment also offers an experimental foundation for the modification research of medical antibacterial protective coatings [7].

#### 2.1.2. Titanium and Titanium Alloys

Titanium and its alloys, especially α+β types such as Ti-6Al-4V [8], are extensively utilized in biomedical applications. These applications encompass orthopedic implants (e.g., hip and knee prostheses), dental implants, and cardiovascular stents. The clinical adoption of these materials is attributed to a distinctive combination of properties, including remarkable biocompatibility, excellent corrosion resistance in physiological environments, low density (approximately 4.5 g/cm^3^), and an elastic modulus (100–110 GPa) that is closer to that of cortical bone (10–30 GPa) compared to traditional implant metals like stainless steel or cobalt–chromium alloys, thus reducing stress shielding. However, their high strength, low thermal conductivity, and chemical reactivity present substantial challenges for conventional machining and welding. To tackle the issue of mechanical mismatch, recent domestic research has focused on the design of metastable β-type titanium alloys with reduced elastic moduli (e.g., Ti-Nb-Zr and Ti-Mo-Sn systems), with the aim of better mimicking the mechanical behavior of bone. Simultaneously, surface engineering, including micro-arc oxidation (MAO), bioactive calcium phosphate coatings, and antibacterial Ag/TiO_2_ nanocomposites, has been demonstrated to be effective in enhancing osseointegration, inhibiting bacterial colonization, and prolonging the service life of implants.

Internationally, 3D printing has brought about a revolutionary change in the development of titanium alloy implants, which enables the creation of patient-specific designs that enhance surgical precision and clinical outcomes. Nevertheless, a crucial limitation persists. Although porous titanium scaffolds fabricated through additive manufacturing can effectively reduce the elastic modulus mismatch and promote initial bone ingrowth, their intrinsic biological inertness results in less-than-optimal osseointegration and weak implant–bone interfacial bonding. To address this challenge, Cheng et al. developed a multifunctional surface modification by applying an atmospheric plasma-sprayed calcium silicate coating co-doped with 2 wt% copper oxide (CuO) and 10 wt% strontium oxide (SrO) on 3D-printed porous titanium scaffolds. In vivo studies using rodent models verified significantly improved bone–implant contact and new bone formation around the modified scaffolds. Complementary in vitro assays further proved potent antibacterial activity against both Gram-positive (*S. aureus*) and Gram-negative (*E. coli*) pathogens, indicating dual functionality: accelerated osseointegration and infection prevention [9].

These implants have progressed from prototype development to regular clinical application in craniofacial and spinal reconstruction. To tackle the fatigue and wear issues related to the long-term performance of titanium alloy implants, which are key factors contributing to aseptic loosening and mechanical failure, researchers across the globe are actively customizing microstructures and improving additive manufacturing and thermomechanical processing techniques. Notably, Xu et al. combined fatigue theory with continuum damage mechanics to explore the high-temperature low-cycle fatigue behavior in Ti_2_AlNb alloy components. Their study indicated that the Extreme Learning Machine (ELM) model surpasses other data-driven methods in predicting fatigue life, providing a reliable and computationally efficient tool for the pre-clinical reliability assessment of next-generation titanium implants [10].

Currently, the majority of modification approaches for titanium and titanium alloys, as well as material optimization strategies, are still limited to laboratory research and pre-clinical validation. A significant challenge that impedes clinical translation is the achievement of cost-effective and scalable manufacturing without sacrificing biosafety. Beyond the progress in conventional fabrication methods, nanostructured titanium alloys, which are engineered through severe plastic deformation or rapid solidification, present a compelling solution. They can simultaneously improve mechanical strength, ductility, and in vitro bioactivity, thus making them promising candidates for the next-generation load-bearing orthopedic and dental implants [11]. Notably, Lv et al. developed a CuO/HA composite doped with Mg, Zn and Ce. The results demonstrated that it exhibited better antibacterial activity against Escherichia coli and Staphylococcus aureus than pure HA. Moreover, CCK-8 assays confirmed that the doped CuO/HA composite had no cytotoxicity and could promote the proliferation of osteosarcoma cells. This study reveals the potential of the material for biomedical applications such as dental implantation and bone regeneration [12].

#### 2.1.3. Cobalt-Based Alloys

The Co-Cr-Mo alloy, a ternary system predominantly composed of cobalt, chromium, and molybdenum, combines outstanding wear resistance, excellent corrosion resistance in physiological environments, and high mechanical strength, rendering it a fundamental material for load-bearing biomedical implants. However, its clinical application is constrained by a crucial biomechanical limitation: its elastic modulus (200–230 GPa) substantially exceeds that of cortical bone (10–30 GPa), resulting in stress shielding, where the implant bears an excessive load, thus inhibiting bone remodeling and potentially causing peri-implant osteoporosis or loosening. Despite this challenge, Co-Cr-Mo continues to be extensively utilized in three critical applications: (1) hip and knee joint replacements, (2) dental prostheses and frameworks, and (3) cardiovascular stents, which have supported decades of surgical progress [13]. To address its drawbacks, research has advanced along two complementary directions. Domestically, efforts are concentrated on compositional refinement (e.g., controlled addition of carbon/nitrogen) and advanced processing (e.g., hot isostatic pressing and selective laser melting) to improve fatigue life and reduce the generation of abrasive wear debris.

Other researchers have developed Ti_3_CT_2x_-UHMWPE nanocomposites to concurrently enhance the mechanical strength, biocompatibility, and biotribological performance of cobalt–chromium alloys. In vitro cytotoxicity assays indicated no adverse impacts on cell viability, which corroborates their biosafety. Notably, compared with unmodified controls, these nanocomposites decreased the coefficient of friction by 19% and the wear rate by 44%, highlighting substantial enhancements in tribomechanical durability [14]. Beyond material modification, additive manufacturing, particularly laser powder bed fusion (LPBF), has facilitated the fabrication of patient-specific orthopedic implants with lattice architectures and functionally graded porosity. Such designs foster osseointegration without undermining structural integrity; several LPBF-fabricated titanium alloy devices have obtained FDA 510(k) clearance and are now regularly utilized in clinical practice [15]. For example, early postoperative outcomes after total ankle arthroplasty with LPBF-optimized pure titanium prostheses demonstrate significant pain reduction and functional improvement, further validating 3D printing as a clinically translatable strategy to enhance both the mechanical and biological functionality of titanium-based implants [16].

#### 2.1.4. Nickel–Titanium Alloy

Nickel–titanium (NiTi), commonly referred to as Nitinol, is a groundbreaking shape-memory alloy that combines the shape-memory effect with superelasticity in a unique manner, allowing for near-complete recovery following significant mechanical deformations. Its remarkable biocompatibility further sets it apart in biomedical applications. It demonstrates negligible cytotoxicity, a minimal immune response, excellent corrosion resistance in physiological environments, and long-term structural stability in vivo. Capitalizing on these advantages, NiTi alloys have become the mainstays in clinical practice, being widely utilized in cardiovascular stents, orthopedic fixation devices for trauma repair, and precision orthodontic archwires [17]. For example, Zhang et al. independently developed a memory-type nickel–titanium alloy wire double-hook device. Experiments have verified that this device can effectively facilitate lacrimal duct stent intubation in patients with lacrimal canalicular laceration [18].

Domestically, research focuses on surface engineering, such as thin-film coatings (e.g., TiO_2_, diamond-like carbon) and composite modifications, to enhance surface bioactivity, reduce the release of nickel ions, and improve wear resistance. Several such advanced stents and orthopedic implants are now in regular clinical use. Internationally, the research frontier has moved towards nickel-free alternatives, especially titanium-niobium (Ti-Nb)-based alloys. Through well-planned alloy design, including the addition of ternary elements (e.g., Zr, Ta) and thermo-mechanical processing, these alloy systems maintain strong shape-memory behavior while eliminating the risks of sensitization associated with nickel. Several Ti-Nb variants have successfully completed pre-clinical safety and efficacy evaluations in large-animal models and have recently entered Phase I/II human clinical trials, which represents a crucial step towards the development of safer, next-generation metallic biomaterials [19].

#### 2.1.5. Magnesium Alloys

This material is predominantly composed of biodegradable magnesium-based alloys, which incorporate essential trace elements such as zinc and calcium. These elements not only support crucial physiological functions, including bone mineralization, enzymatic activity, and cellular signaling, but also enhance the alloy’s inherent biocompatibility. A significant advantage is its controllable biodegradation: it gradually decomposes in physiological environments, releasing non-toxic ions that can be safely metabolized or excreted. This process minimizes inflammatory responses and avoids long-term foreign-body complications. However, the relatively rapid corrosion of this material in chloride-rich biological fluids, such as blood and interstitial fluid, remains a challenge. This may potentially lead to premature mechanical failure before tissue healing is completed. To tackle this issue, current research focuses on two synergistic strategies: (1) compositional optimization through targeted alloying (for example, adding rare-earth elements like yttrium or zinc to refine the microstructure and suppress localized corrosion), and (2) functional surface engineering, including bioceramic coatings (such as hydroxyapatite) and polymer-based barriers, to precisely regulate degradation kinetics and improve interfacial tissue integration. Consequently, these advanced magnesium alloys are now being applied in clinical settings: they serve as next-generation absorbable orthopedic implants (such as load-bearing bone plates and compression screws) and are promising candidates for fully bioresorbable cardiovascular stents, providing temporary scaffolding without the need for secondary removal. In China, national research and development (R&D) initiatives have expedited progress in this field. Multiple alloy systems, such as Mg-Zn-Ca and Mg-Y-Zn, have advanced to pre-clinical and early clinical trials, especially for orthopedic and cardiological indications. Internationally, magnesium alloys are increasingly being adopted in clinical applications, particularly in cardiovascular medicine. Several degradable magnesium-alloy stents have progressed to human clinical trials. The early-phase results suggest excellent biocompatibility, predictable and adjustable degradation kinetics, and significant translational potential for real-world patient care [20].

The medical metal materials industry is confronted with persistent challenges that impede its clinical translation and long-term sustainability. Despite the consistent progress achieved in material innovation, the widespread adoption of medical metal materials remains restricted by three interrelated bottlenecks: (1) a fragmented technological ecosystem, in which fundamental research, process engineering, and regulatory validation function in isolation; (2) slow commercialization, where only a small proportion of promising lab-scale alloys progress to Good Manufacturing Practice (GMP) manufacturing and clinical trials; and (3) processing complexity. High-performance metals such as titanium, nickel–titanium, tantalum, and zirconium require energy-intensive and precision-dependent fabrication methods (e.g., powder metallurgy, additive manufacturing, or severe plastic deformation), which increase the cost and pose barriers to scalability. Beyond the aspect of manufacturability, biological performance is of crucial importance. Conventional implants, such as 316L stainless steel and Co-Cr-Mo alloys, present dual biocompatibility risks. Metal ion leaching can lead to local inflammation, hypersensitivity, or systemic immune activation. Simultaneously, the mismatch in elastic modulus (e.g., stainless steel: ~200 GPa vs. cortical bone: 10–30 GPa) induces stress shielding, which suppresses bone remodeling, delays osseointegration, and elevates the risk of aseptic loosening. Even after successful implantation, the long-term functionality of implants is affected by in vivo degradation mechanisms. Cyclic loading generates wear debris, which fuels chronic peri-implant inflammation and osteolysis. Fatigue accumulation undermines the mechanical integrity of the implants, leading to microfractures, pain, and ultimately, revision surgery, which burdens patients physically, psychologically, and financially. Finally, diagnostic compatibility should not be overlooked: ferromagnetic stainless steels cause distortion of MRI fields, whereas high-Z elements (e.g., Ta, Zr) generate streaking artifacts in CT and radiography, which hinders postoperative monitoring and early complication detection.

In light of the rapid progress in bimetallic and advanced metallic materials, a new generation of biomedical metals, including powder metallurgy alloys, high-entropy alloys, metallic glasses (amorphous alloys), and liquid-phase nanostructured metals, has come into existence. These materials not only surpass the conventional mechanical performance but also possess inherent biological functionalities, such as antimicrobial activity, selective antitumor effects, and immunomodulatory or osteoinductive behavior. To further enhance the biofunctionality without undermining the structural integrity, surface modification, through physical methods (e.g., plasma spraying, laser texturing) or chemical methods (e.g., silanization, peptide grafting), remains the most commonly employed strategy. It precisely modifies the surface topography, chemistry, and energy while maintaining the bulk material’s strength, ductility, and fatigue resistance [21]. Simultaneously, 3D-printed titanium alloy implants have shifted from laboratory research to the initial stage of clinical application, especially in load-bearing orthopedic and dental reconstructions [22]. In line with this trend, metal-based functional gradient materials (FGMs) have attracted considerable attention. Recent studies indicate that optimized FGMs can improve surface corrosion resistance by approximately 154% compared to homogeneous counterparts. They can also maintain uniform mechanical integrity across interfaces and significantly enhance biocompatibility, bone-bonding capacity (osseointegration), and stem-cell-driven osteogenic differentiation, which makes them particularly promising for temporary, load-adaptive orthopedic implants [23].

AI-driven innovations in metallic biomaterials are expeditiously revolutionizing biomedical research and clinical practice. In 2021, a significant study conducted by a prominent international team presented enzyme-powered liquid metal nanorobots that can autonomously navigate along urea gradients, thereby facilitating precise, on-demand drug delivery and synergistic antibacterial therapy in intricate physiological environments. Capitalizing on this advancement, FDA-approved nanometallic therapeutics, such as iron oxide nanoparticles for MRI contrast and gold-based agents for photothermal tumor ablation, have already been put into clinical use [24]. Most recently, Yang et al. (2023) introduced sub-3 nm minted metal nanoclusters as dual-modal theranostic platforms, systematically resolving critical bottlenecks in biocompatibility, targeting fidelity, and real-time monitoring [25]. Looking forward, the integration of AI-guided design, high-resolution 3D bioprinting, and dynamic surface engineering not only holds the promise of enhanced functionality but also offers unprecedented control over the safety, specificity, and scalability of next-generation metallic medical devices.

### 2.2. Medical Polymer Materials: Key Carriers for Functional Diversity

Biomedical polymer materials, which are highly regarded for their biocompatibility, low toxicity, and structural resemblance to native human tissues, play a crucial role in modern medicine. They support critical applications such as tissue engineering, controlled drug and gene delivery, implantable medical devices, point-of-care diagnostics, and antimicrobial (antiviral and antibacterial) therapies. The key material classes encompass nanoparticles, hydrogels, stimulus-responsive polymers, and implantable scaffolds. Propelled by the rapid progress in biotechnology and clinical requirements, research is increasingly centered on improving functionality, biodegradability, spatiotemporal responsiveness, and biological fidelity. The major categories of biomedical polymers cover both synthetic and natural origins, including biodegradable polymers (e.g., PLGA, PCL), hydrogels (natural and synthetic), antimicrobial polymers, tissue-engineered scaffolds, targeted drug delivery carriers, biomimetic polymers, and conductive polymers.

#### 2.2.1. Biodegradable Polymeric Substances

Biodegradable polymers are comprehensively classified into two primary categories: natural and synthetic. Natural biopolymers, such as cellulose derivatives (e.g., carboxymethyl cellulose), collagen, and chitosan, present distinctive biological compatibility and adjustable degradation profiles. For instance, oxidized regenerated cellulose (ORC) is extensively utilized in surgical hemostasis because of its outstanding adhesion to wet tissues and rapid absorption; it completely degrades within 10–14 days without the need for removal. Likewise, the reversible thermosensitivity of collagen, which remains liquid below 25 °C and forms a stable hydrogel at physiological temperature (37 °C), renders it particularly suitable for minimally invasive subcutaneous delivery and soft-tissue reconstruction in plastic surgery [26].

Natural materials are characterized by their abundance and biocompatibility; however, precisely tailoring their mechanical properties and degradation rates poses significant challenges. In contrast, synthetic biodegradable polymers, such as polylactic acid (PLA), polyhydroxyalkanoates (PHA), and polycaprolactone (PCL), provide enhanced control over performance and degradation behavior. PLA, which is obtained through the polymerization of lactic acid, degrades in soil within a period of 6 months to 1 year and is extensively utilized in compostable packaging and orthopedic fixation devices. PHA, produced naturally via microbial fermentation, is fully biodegradable under a variety of environmental conditions and shows great potential for medical implants and sustainable bioplastics. PCL demonstrates a slower degradation rate (typically 1–2 years), rendering it suitable for long-term drug delivery systems and tissue engineering scaffolds. The degradation of these polymers mainly occurs through three pathways: (1) enzymatic hydrolysis (for example, PLA is cleaved into lactic acid monomers, which are then metabolized to CO_2_ and H_2_O); (2) photodegradation (light-induced bond scission); and (3) thermal degradation (bond cleavage triggered by elevated temperatures). Notably, although photodegradation and thermal degradation can facilitate accelerated breakdown, they may undermine the structural integrity or produce unintended by-products. Therefore, controlled enzymatic hydrolysis remains the most physiologically relevant and predictable route for biomedical applications.

The performance of degradable polymer materials is precisely adjusted through three interrelated strategies: controlled degradation, customized mechanical behavior, and improved thermal stability. Firstly, the degradation kinetics can be reasonably regulated via copolymerization (e.g., adjusting the LA:GA ratio in PLGA), surface functionalization, or blending with natural or synthetic polymers, which enables time-matched scaffold resorption and tissue regeneration. This is demonstrated by human dental pulp stem cells showing robust proliferation and multilineage differentiation within PLGA scaffolds [27]. Secondly, the mechanical properties are engineered to meet the specific requirements of applications. Chemical modification (e.g., etherification or esterification) significantly enhances the tensile strength of cellulose derivatives, while PLA-PCL blends synergistically improve toughness without sacrificing biodegradability. Thirdly, thermal stability is optimized not only by slowing down decomposition but also by strategically incorporating nanofillers (e.g., surface-modified carbon nanotubes) or introducing covalent cross-links. This ensures the processing integrity during melt extrusion or electrospinning while maintaining physiological degradation profiles [28].

Biodegradable polymer materials can be fabricated through three primary approaches: physical processing, chemical synthesis, and polymer blending. Physical methods, such as solution casting and melt compression, are operationally straightforward and cost-efficient, which makes them suitable for large-scale production of packaging films. Nevertheless, they often result in materials with restricted mechanical strength and thermal stability. In contrast, chemical methods, including ring-opening polymerization (for example, for polylactic acid, PLA) and controlled cross-linking, allow for precise molecular design and the formation of high-purity products, thus improving functional performance. Polymer blending (e.g., PLA/PCL blends) provides a practical strategy to synergistically combine complementary properties. However, thermodynamic immiscibility often causes phase separation, a challenge that is being actively tackled through compatibilizers, nanostructuring, or reactive blending techniques.

Biodegradable polymer materials are assuming an increasingly significant role in orthopedic applications, encompassing absorbable sutures, bioresorbable bone fixation devices, and advanced regenerative scaffolds. In contrast to traditional implants, these materials gradually degrade in vivo into non-toxic byproducts, obviating the necessity for secondary removal surgery and consequently reducing both clinical risks and healthcare costs. Beyond providing structural support, biodegradable polymers like PLGA function as intelligent drug carriers. Their nanoparticles facilitate spatiotemporally controlled, pathogen-targeted delivery, enhancing therapeutic efficacy while minimizing off-target effects and systemic toxicity [29]. Moreover, the integration of 3D-printed bionic scaffold technology has transformed tissue engineering, enabling the precise replication of native bone and cartilage microarchitectures. This confluence of material design, controlled degradation, and additive manufacturing is now enabling clinical solutions for previously intractable musculoskeletal conditions [30].

Biodegradable polymer materials present a transformative potential in the domains of environmental sustainability and biomedical innovation. However, realizing their full potential depends on surmounting two persistent bottlenecks: the trade-off between degradation kinetics and mechanical integrity, and the scalability-cost barrier. Take the well-known dichotomy as an example: polycaprolactone (PCL) exhibits excellent tensile strength but degrades at a pace too slow for numerous clinical applications; polylactic acid (PLA), despite its rapid degradability, is characterized by inherent brittleness and poor toughness. These limitations are not insurmountable obstacles but rather design challenges that can be addressed through rational engineering. Recent advancements indicate that strategic copolymerization (e.g., PLA-PCL block copolymers), controlled blending with plasticizers or nanoreinforcements, and stimuli-responsive surface functionalization allow for the precise adjustment of both degradation profiles and mechanical behavior. At the domestic level, research has advanced from simple synthesis to structure–property–function mapping, as evidenced by tunable PLGA scaffolds achieving matched degradation rates and neo-tissue formation in preclinical bone regeneration models. On a global scale, the field is rapidly moving towards translation: fully bioresorbable PLGA-based coronary scaffolds have successfully completed Phase III trials, showing non-inferiority to metallic stents in terms of safety and efficacy, with complete scaffold resorption occurring within 24 months [31].

Degradable materials function as highly versatile drug carriers for small-molecule drugs, proteins, and nucleic acids. Once implanted or administered, they experience controlled enzymatic or hydrolytic degradation in the in vivo environment, facilitating the sustained and adjustable release of therapeutics. This, in turn, extends the therapeutic exposure period while minimizing systemic toxicity. Significantly, this approach alleviates the risks related to burst release (such as acute toxicity and off-target effects), reduces the frequency of dosing, and enhances patient compliance with treatment regimens. Through the rational design of the monomer composition, molecular weight, crystallinity, and architecture of degradable polymers (for example, PLGA, PCL, and PEG-based copolymers), researchers can accurately customize the degradation kinetics and drug release profiles to meet physiological requirements, enabling spatiotemporally controlled delivery to disease sites. This enhances drug accumulation at the lesion site, minimizes collateral damage to healthy tissues, and increases the therapeutic index. These benefits have promoted their application in oncology (such as localized chemotherapy and immunomodulator delivery) and chronic disease management (such as long-term hormone or biologic therapy). A typical example is the research conducted by Obayemi et al., who fabricated porous, FDA-compliant scaffolds by blending three clinically established polymers: PCL, PEG, and PLGA. Comprehensive physicochemical characterization (including SEM, DSC, GPC, and degradation kinetics assays) revealed the structure–property relationships that govern scaffold stability and drug release. Subsequent in vitro release studies under physiological temperatures (37 °C) and hyperthermic conditions (42 °C), combined with murine orthotopic mammary tumor models, demonstrated not only the sustained release of doxorubicin but also significant tumor growth inhibition and concurrent regeneration of adjacent normal mammary tissue [32].

Looking forward, the integration with computational polymer design, enzyme-triggered degradation systems, and solvent-free melt-processing technologies will be crucial, not only to enhance performance but also to enable reliable, reproducible, and economically viable manufacturing for regenerative medicine, sustainable packaging, and other related fields.

#### 2.2.2. Hydrogel Materials

Hydrogels have emerged as a fundamental material in biomedical applications, due to their outstanding biocompatibility, high water absorption and retention capabilities, as well as the structural and functional resemblance to the native extracellular matrix (ECM). Their versatility allows for targeted therapeutic functions across multiple clinical fields: (1) Bone regeneration—hydroxyapatite-reinforced hydrogels offer osteoconductive scaffolds that actively facilitate mineral deposition and new bone formation [33]; (2) Articular cartilage repair—cellulose-based hydrogels mimic the compressive resilience and low-friction biomechanics of native cartilage, supporting chondrocyte proliferation and extracellular matrix synthesis [34]; (3) Accelerated wound healing—chitosan-based hydrogels integrate high hydration, oxygen permeability, and inherent antibacterial activity to maintain a moist, infection-resistant healing microenvironment; and (4) Spatiotemporally controlled drug delivery—intelligent stimuli-responsive hydrogels (e.g., NIPAM-PMAA copolymers) enable the precise, on-demand release of therapeutics in response to pathological signals such as tumor-specific pH or localized hyperthermia, thereby enhancing treatment efficacy while minimizing off-target toxicity [35].

Biodegradable hydrogels, which are highly regarded for their flexibility, biocompatibility, and tissue-mimetic mechanical behavior, are emerging as promising candidates for heart valve repair. However, despite their extensive potential in regenerative medicine, drug delivery, and biosensing, the clinical translation of these hydrogels is still impeded by four persistent challenges: (1) Insufficient mechanical robustness, especially under cyclic physiological loads, which results from the low cross-linking density in natural polymer-based systems (e.g., chitosan, cellulose); (2) poorly adjustable degradation kinetics, as in vivo factors such as local pH, enzymatic activity, and inflammatory status lead to unpredictable erosion rates that affect the therapeutic timing; (3) residual immunogenicity or cytotoxicity, particularly associated with certain synthetic monomers or cross-linkers that cannot be completely removed during purification; and (4) scalability bottlenecks, as lab-scale fabrication methods, including UV-initiated photopolymerization and gamma irradiation, lack reproducibility, throughput, and regulatory compatibility for GMP-compliant manufacturing. Recent progress in dual-network design, nanocomposite integration, and reversible dynamic bonds has started to tackle the first challenge. Similarly, enzyme-responsive moieties and microenvironment-sensitive linkers provide new means for degradation control. Nevertheless, to bridge the gap between bench innovation and bedside application, integrated optimization is required, not only of individual material properties but also of manufacturability, sterility assurance, and long-term functional stability in dynamic cardiac environments.

In response to these challenges, researchers across the globe are making consistent progress by driving innovation through three key strategies: (1) rational structural design, (2) advanced fabrication methods, and (3) targeted functionalization. Nationally, studies predominantly concentrate on biocompatible hydrogels, including both natural (e.g., hyaluronic acid, chitosan) and synthetic (e.g., polyethylene glycol, PEG) ones, which have well-established applications in wound dressings, controlled drug delivery, and tissue engineering scaffolds. To enhance mechanical robustness and bioactivity, researchers utilize customized cross-linking chemistries and site-specific functional modifications. Internationally, stimuli-responsive “smart” hydrogels, especially those sensitive to pH, temperature, or enzymatic cues, are emerging as next-generation platforms for spatiotemporally controlled drug release and dynamic tissue regeneration.

The performance of hydrogels can be significantly enhanced through strategic design across multiple scales. At the molecular level, dynamic covalent bonds facilitate reversible cross-linking and self-healing [36]. At the microstructural level, a controlled pore architecture, which is achieved via directional freezing, Hofmeister-series ion modulation, or co-solvent templating, improves water permeability and drug loading capacity. At the biomimetic level, decellularized extracellular matrix (dECM)-based hydrogels replicate native tissue cues to support cell adhesion and regeneration [37]. Functionality can also be adjusted through stimuli-responsiveness. For example, poly(N-isopropylacrylamide) (PNIPAM)-based hydrogels experience reversible sol–gel transitions near physiological temperature, which enables their applications in ocular delivery and minimally invasive wound sealing [38]. Similarly, Yu et al. used thermosensitive hydrogels to prolong the retention of extracellular vesicles derived from human umbilical cord mesenchymal stem cells in the uterine environment, thereby improving the therapeutic efficacy against intrauterine adhesions [39]. Meanwhile, salt-induced phase separation has been utilized to fabricate chitosan/gelatin/albumin composite dressings with adjustable mechanical integrity and bioactivity [40].

As materials science and engineering technologies progress, hydrogels, which are highly biocompatible, water-swelling polymers with adjustable 3D network structures, are rapidly extending beyond their traditional biomedical applications. Having already played a crucial role in drug delivery and tissue engineering, they are currently attracting attention in environmental remediation (e.g., heavy-metal adsorption and wastewater filtration), next-generation energy storage (e.g., stretchable supercapacitors), and soft electronics (e.g., wearable biosensors). Their versatility is derived not only from their outstanding water-retention ability but also from their programmable mechanical, electrical, and responsive properties, which makes them a fundamental material for interdisciplinary innovation.

#### 2.2.3. Antibacterial Polymer Materials

Antibacterial polymer materials are functional polymers that are engineered through chemical synthesis or physical modification to provide persistent, stimuli-responsive, or on-demand antibacterial activity. Their mechanisms can be classified into three main categories: (1) direct killing of bacteria through membrane disruption or metabolic interference; (2) suppression of bacterial proliferation by inhibiting biofilm formation or quorum sensing; and (3) sustained release of embedded antimicrobial agents for long-term protection. Based on their origin, these materials are generally classified as natural (e.g., chitosan, lignin) or synthetic (e.g., quaternized polyethylenimine, antimicrobial peptide-polymer conjugates), with each type balancing processability, biocompatibility, and efficacy. However, the practical application of these materials faces significant challenges: unmodified biopolymers such as cellulose often lack inherent activity and require expensive functionalization, while advanced systems, such as metal-or graphene oxide-based nanocomposites, are limited in scalability due to complex, multi-step syntheses and poor batch-to-batch reproducibility.

Domestic research has achieved remarkable progress in the development of antibacterial polymer materials for biomedical applications, especially in medical devices and wound dressings. The key strategies encompass chemical modification (e.g., grafting of quaternary ammonium salts), incorporation of antimicrobial agents at the nanoscale (e.g., silver nanoparticles), and bioinspired design utilizing natural antimicrobial peptides or cationic polymers such as polylysine. These approaches not only augment broad-spectrum antibacterial activity, including against drug-resistant strains, but also enhance biocompatibility and functional stability through controlled surface engineering and hierarchical nanostructure assembly. Significantly, polylysine-antimicrobial peptide conjugates have exhibited synergistic bactericidal effects in pre-clinical studies, which lays the foundation for clinical translation [41]. In dentistry, enamel-mimicking polymer composites represent another area of exploration: by replicating the hierarchical mineral-polymer architecture of natural teeth through biomimetic mineralization, they substantially improve the durability of restorations and long-term integration with host tissue [42].

#### 2.2.4. Biomimetic Polymer Materials

Biomimetic polymer materials are functional polymers that draw inspiration from nature. They are designed by emulating the hierarchical structures, dynamic functions, or biosynthetic strategies of living organisms. The primary objective of these materials is to endow them with exceptional mechanical performance, environmental responsiveness, and biocompatibility by accurately replicating biological design principles. These principles range from nanoscale motifs (e.g., collagen triple helices) and microscale architectures (e.g., nacre’s brick-and-mortar layers) to macroscale systems (e.g., vascular networks in bone), as well as adaptive behaviors such as self-healing, stimulus-triggered deformation, and autonomous self-regulation. Specific examples include superhydrophobic coatings inspired by lotus leaves, polyamide fibers mimicking spider silk that exhibit both high tensile strength and reversible hydrogen-bond-mediated self-repair [43], and composites inspired by nacre that combine ceramic platelets with polymer interlayers to achieve unprecedented toughness [44]. At their core, these materials are interdisciplinary, bridging polymer chemistry, materials engineering, and systems biology. They have already facilitated advancements in regenerative medicine (e.g., low-immunogenicity collagen hydrogels for tissue scaffolds), sustainable energy (e.g., ion-selective membranes mimicking biological channels), environmental remediation (e.g., enzyme-integrated hydrogels for pollutant degradation), and adaptive electronics (e.g., stretchable, self-healing conductors). However, significant challenges persist, such as achieving atomic-level precision in hierarchical assembly, ensuring long-term structural integrity under physiological or operational stress, and scaling up fabrication without sacrificing biomimetic fidelity. Addressing these challenges demands not only advanced synthesis and characterization tools but also a more profound integration of biological insight with engineering design logic.

**Figure 1:**
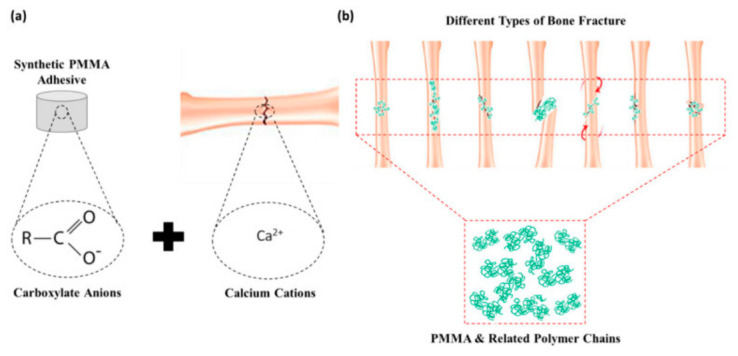
Mechanism of PMMA-based adhesives: (**a**) chemical/physical bonding via carboxylate-Ca^2+^ ionic interactions; (**b**) mechanical interlocking via polymer chain penetration into surface irregularities [45].

Laboratory-scale fabrication methods, especially 3D-printed bionic scaffolds, encounter challenges in scaling up for industrial applications because of limitations in equipment capacity, process reproducibility, and throughput. Significantly, the hierarchical complexity of native tissues, such as multi-scale porosity, spatially graded mechanics, and dynamically reversible cross-linking, is still difficult to reproduce accurately, thus restricting functional performance and clinical translation. As shown in Figure 1, Tzagiollari’s group is at the forefront of developing eco-degradable, physiologically compatible surgical adhesives that are specifically engineered for load-bearing bone fixation, accelerated fracture healing, and guided osteo-regeneration [45]. Meanwhile, Li Haoyi’s team is making progress in key enabling technologies, including polymer melt electrospinning (with novel nozzle designs and real-time fiber morphology control), centrifugal spinning systems for high-throughput microfiber production, micro/nano-filtration membranes with tunable pore architecture, 3D bioprinting platforms for spatially resolved scaffold fabrication, and transdermal microneedle patches for programmable drug release. Globally, researchers are increasingly combining biomimetic hydrogels with multi-material bio-3D printing to fabricate perfusable vascularized tissue constructs, which represents tangible progress towards functional organ surrogates [46]. In the future, the integration of bionic design principles, adaptive materials chemistry, and intelligent manufacturing will propel next-generation biomaterials towards precision regenerative therapies, sustainable medical devices, and energy-efficient biomedical interfaces.

#### 2.2.5. Conductive Polymer Materials

Conductive polymers represent a distinct category of plastics capable of conducting electricity; some of them even exhibit conductivity comparable to that of metals or semiconductors. In contrast to traditional insulating polymers, the electrical activity of conductive polymers stems from their extended π-conjugated backbones, which facilitate the delocalization of electrons along the polymer chain. Conductivity is not an inherent property but is activated via doping, either chemically (e.g., using FeCl_3_ or HCl) or electrochemically, which introduces charge carriers such as polarons or bipolarons. These carriers enable efficient charge transport, thereby converting the material from an insulator into a functional conductor [47].

Conductive polymer materials present a highly appealing combination of distinctive advantages for next-generation electronic and biomedical applications. Firstly, their extremely low density, which is merely one-fifth to one-tenth of that of conventional metals, renders them suitable for lightweight, portable, and wearable devices. Secondly, they can be highly processed through scalable, low-energy techniques such as solution casting, inkjet printing, and electrochemical deposition, facilitating the easy fabrication of flexible, stretchable, and patterned architectures. Thirdly, their electrical conductivity ranges across an extraordinary 15 orders of magnitude, from 10^−10^ to 10^5^ S/cm, and can be precisely adjusted through rational doping strategies and molecular engineering of the conjugated backbone [48]. Fourthly, in contrast to most metals, conductive polymers display remarkable chemical stability in harsh acidic and alkaline environments, providing superior corrosion resistance that is crucial for long-term operation in biosensors and implantable devices. Finally, apart from conductivity, they incorporate multifunctionality: the reversible redox activity of polyaniline enables high-performance supercapacitors; polythiophene derivatives exhibit a strong photoelectric response; certain copolymers offer effective electromagnetic interference (EMI) shielding; and several systems demonstrate promising thermoelectric conversion efficiency. Collectively, these characteristics position conductive polymers not only as alternatives to metals but also as intelligent, designable functional platforms at the intersection of materials science, electronics, and biomedicine. Composite materials: Key advantages and limitations of conductive polymers. Composite conductive polymers, such as carbon black-reinforced silicone rubber, integrate the elasticity and toughness of the polymer matrix with the electrical functionality of conductive fillers [49]. This combination results in distinct advantages, including a low electrical percolation threshold (frequently below 3 vol%), inherent antistatic performance, and effective electromagnetic interference (EMI) shielding across a wide range of frequencies.

However, traditional intrinsically conductive polymers, including polyaniline (PANI) and polypyrrole (PPy), encounter significant practical limitations [50]. Firstly, their rigid, conjugated backbones impede processability. They display poor solubility and minimal melt viscosity, making standard thermoplastic processing (e.g., extrusion or injection molding) unfeasible without chemical modification. For example, PANI requires protonic acid doping or surfactant-stabilized emulsion polymerization to achieve dispersibility in common solvents. Secondly, environmental stability remains a crucial challenge. Prolonged exposure to UV light, high temperatures, or mechanical cycling causes irreversible dedoping and chain scission, leading to a rapid decline in conductivity. For instance, PPy’s conductivity can decrease by more than 50% within 72 h in ambient humid air (60% RH, 25 °C). Thirdly, their mechanical integrity is inherently weak. Pure PPy films have tensile strengths below 2 MPa, which is orders of magnitude lower than those of structural polymers. Therefore, reinforcement through blending (e.g., with polyvinyl alcohol) or nanocomposite design is necessary to meet application requirements.

The underlying conduction mechanism remains inadequately understood, especially the interaction among percolation theory, quantum tunneling, and field-induced electron emission. Meanwhile, microcracks inevitably disrupt conductive pathways, necessitating self-healing functionality. Incorporating dynamic covalent or non-covalent bonds (such as Diels-Alder adducts, hydrogen bonds, or metal-ligand coordination) presents a promising strategy. Moreover, scalable manufacturing is hampered by synthetically challenging routes. For example, chiral mesoporous polypyrrole, a representative nanostructured conductive polymer, requires multi-step templating, strict environmental control, and costly reagents, which impedes low-cost, high-yield production.

**Figure 2:**
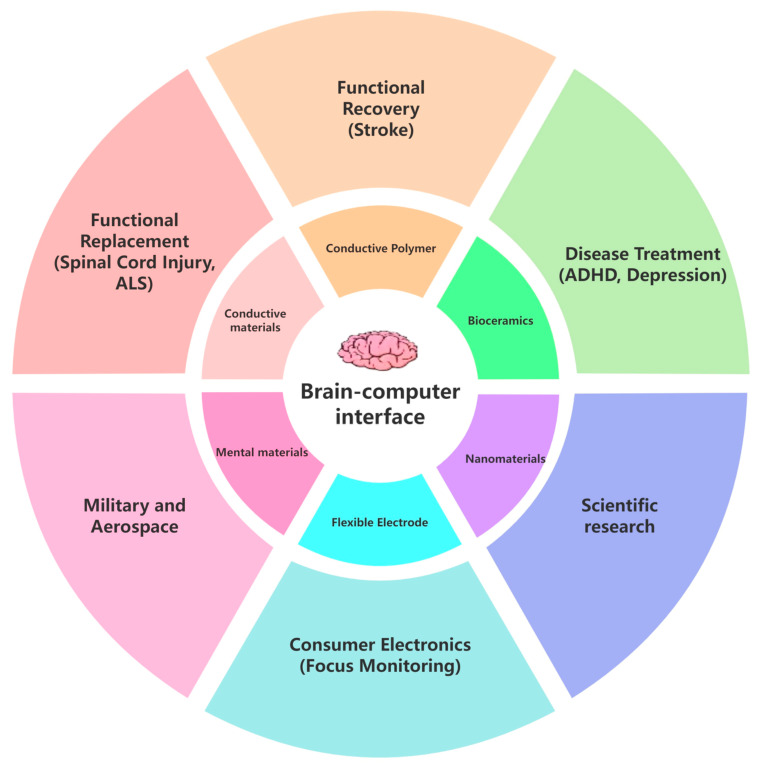
Core areas and application materials of brain–computer interfaces.

Domestic research has achieved significant progress in the development of conductive polymer materials, such as polyaniline, polypyrrole, and polythiophene, for neural electrodes and biosensors. In order to improve both electrical conductivity and biocompatibility, researchers are increasingly adopting nanotechnology and composite material strategies [51]. Remarkable advancements have been made in nerve regeneration therapies and next-generation wearable health monitors, with several prototypes currently entering clinical trials. For example, nanostructured polyaniline-based electrodes are being assessed in preclinical brain–computer interface systems (Figure 2) [52]; meanwhile, a highly selective electrochemical immunosensor, constructed from conductive carbon black and star-shaped poly(glycidyl methacrylate) (PGMA), enables the sensitive detection of interleukin-8 (IL-8) in human serum and saliva [53].

Conductive polymer materials are undergoing rapid evolution towards multifunctionality, intelligence, and sustainability. In order to realize this potential, researchers need to surmount fundamental bottlenecks, especially in comprehending charge transport mechanisms, and initiate innovative doping approaches, such as ionic liquid-mediated doping [54]. Equally crucial is the synergistic integration of nanostructured conductive polymers with flexible electronics. Leveraging international advancements in wearable electronics and green material design, China’s research community should enhance industry-university-research collaboration to expedite scalable fabrication and real-world implementation.

#### 2.2.6. Natural Polymer Materials

Natural polymer materials are macromolecular compounds that are either directly extracted from living organisms or biosynthesized by microorganisms or plants. Owing to their inherent biocompatibility, biodegradability, and bioactivity, they have become essential in biomedicine, food science, agriculture, and environmental remediation. Notable examples include tannic acid, a plant-derived polyphenol featuring multiple phenolic hydroxyl and carboxyl groups, which facilitate strong hydrogen bonding and metal chelation. It is extensively utilized as a natural antioxidant and antimicrobial agent in functional foods and cosmeceuticals. Sodium alginate, an anionic linear polysaccharide obtained from brown algae through alkaline extraction, forms reversible ionotropic gels (e.g., with Ca^2+^), rendering it highly valuable for encapsulation, thickening, and wound dressing applications in the food, pharmaceutical, and agricultural sectors [55]. Chitosan, produced by the partial deacetylation of chitin from crustacean shells or fungal mycelia, demonstrates pH-responsive solubility, mucoadhesiveness, and inherent antimicrobial activity. It is clinically employed in hemostatic sponges, 3D-bioprinted scaffolds for tissue regeneration, and controlled-release nanocarriers for therapeutics [56]. Despite these benefits, natural polymers encounter persistent challenges, such as inherently low mechanical strength (e.g., tensile modulus often <100 MPa), vulnerability to enzymatic or hydrolytic degradation under physiological conditions, and processing limitations like thermal instability and poor melt processability. These constraints prompt continuous research into physical blending, chemical crosslinking, and nanocomposite reinforcement.

**Figure 3:**
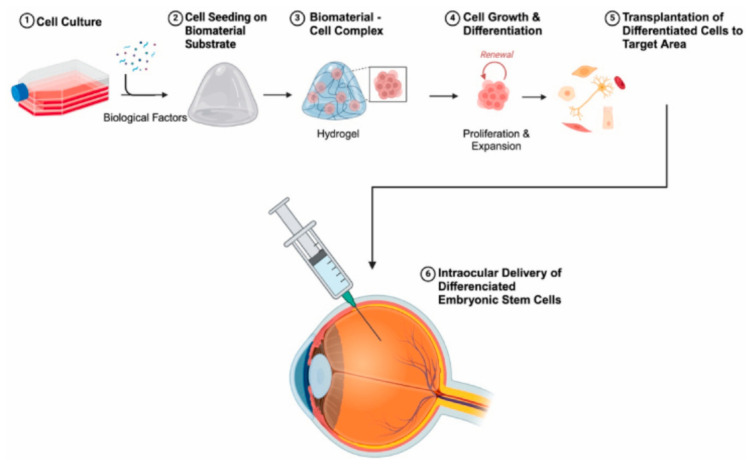
Application of hydrogel in stem cell therapy. Reproduced with permission [58].

Current research on natural polymers predominantly focuses on chemical modification and composite strategies to enhance their mechanical strength, bioactivity, and functional responsiveness, which are key requirements for advanced biomedical applications. Chitosan, hyaluronic acid, collagen, and cellulose are among the most extensively studied candidates. For instance, sodium alginate-chitosan microspheres notably improve drug bioavailability via controlled encapsulation and pH-responsive release. Meanwhile, smart hydrogels, which are engineered to respond to stimuli such as temperature, pH, or enzymes, enable spatiotemporally precise drug delivery. Beyond drug delivery, these materials contribute to the progress in regenerative medicine. Hydroxyapatite-collagen scaffolds imitate the native bone extracellular matrix to expedite osteogenesis, and gellan gum-based hydrogels offer biomimetic 3D microenvironments that support stem cell adhesion, proliferation, and differentiation (Figure 3) [57,58]. These advanced biomaterials show significant potential for skin regeneration, especially bacterial cellulose-based dressings, which not only accelerate wound healing but also function as smart, targeted drug delivery systems [59]. Beyond biomedical applications, they are promoting innovation in sustainable manufacturing (e.g., enzyme-or microbe-mediated biosynthesis substituting energy-intensive chemical modifications) and responsive functional design (e.g., 4D-printed materials that dynamically adapt to physiological cues).

Natural polymer materials encompass a diverse array of biomacromolecules. Among them, silk-based proteins, specifically fibroin, sericin, and spider silk spidroins, are notable for their outstanding structural and functional properties. Silk fibroin, which is abundant in glycine and alanine, self-assembles into stable β-sheet nanofibrils, endowing it with remarkable tensile strength and toughness. In contrast, sericin demonstrates strong hydrophilicity, excellent biocompatibility, and controllable biodegradability, rendering it suitable for biomedical coatings and drug carriers. Spider silk proteins, especially major ampullate spidroins (MaSp1 and MaSp2), combine ultra-high strength with extraordinary elasticity, comparable to synthetic high-performance fibers. Owing to advancements in recombinant expression systems, these proteins can now be produced on a large scale without sacrificing their native mechanical integrity or biological functionality. They can be easily processed into various forms, including fibers, thin films, injectable hydrogels, porous 3D scaffolds, and nanocomposites. Their performance can be precisely adjusted by blending with natural or synthetic polymers or functional inorganic nanoparticles (e.g., hydroxyapatite). Consequently, silk-derived materials have emerged as enabling platforms in tissue engineering, controlled drug delivery, flexible bioelectronics, and regenerative medicine. For example, micro-patterned spider silk substrates presenting RGD motifs significantly enhance the adhesion and spontaneous chondrogenic differentiation of human Wharton’s jelly mesenchymal stem cells (hWJ-MSCs), even in the absence of exogenous growth factors, which leads to accelerated bone defect regeneration [60]. Moreover, spider silk-based electroactive dressings generate endogenous electrical cues that accelerate wound closure, particularly in challenging joint injuries [61]. These dressings also facilitate skin regeneration and have been effectively applied in high-end cosmetic applications, such as restorative facial masks and bioactive hair care formulations [62]. Significantly, the integration of genetic engineering and nanotechnology now allows for precise and programmable control over the material’s structure, mechanical properties, and biological functionality, thus introducing a new generation of smart biomaterials for regenerative medicine and environmental remediation [63].

### 2.3. Medical Composites: An Innovative Approach for Multi-Performance Synergy

**Figure 4:**
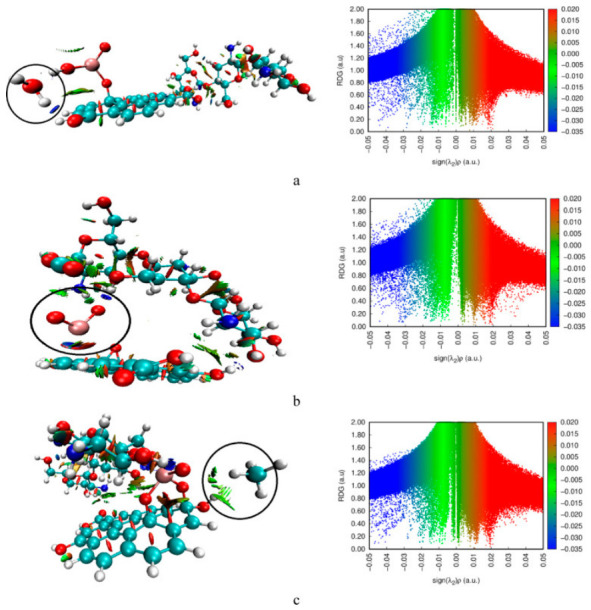
Calculated non-covalent Interaction (NCI) for the Cs/GO/TiO_2_ nanocomposite interacting with gas molecules, specifically: (**a**) Cs/GO/TiO_2_/H_2_O; (**b**) Cs/GO/TiO_2_/CO_2_ and (**c**) Cs/GO/TiO_2_/CH_4_ [64].

Medical composites are advanced biomaterials that are engineered by integrating two or more distinct constituent materials, such as polymers, metals, and ceramics, via physical or chemical strategies. These composites are designed to synergize the complementary advantages of each phase, thereby meeting the strict functional requirements in clinical settings. Their key characteristics encompass excellent biocompatibility, adjustable mechanical properties (e.g., stiffness, strength, and degradation rate), and multifunctionality, such as inherent antibacterial activity, osteoinductive capacity, or stimuli-responsive behavior. For instance, chitosan/graphene oxide (CS/GO) composites utilize chitosan’s bioactivity and antimicrobial action in conjunction with graphene oxide’s outstanding mechanical reinforcement and electrical conductivity (Figure 4) [64]. Likewise, nanocrystalline cellulose-reinforced chitosan hydrogels display shear-thinning rheology, shape fidelity, and cytocompatibility, which makes them highly appropriate for extrusion-based 3D bioprinting of tissue-engineered constructs. Moreover, surface functionalization, such as calcium phosphate coating or RGD peptide grafting, facilitates the integration of scaffolds with native bone tissue, thus enhancing osteointegration and supporting regenerative therapies beyond orthopedics, including wound healing and drug-eluting implants [65].

Medical composites, which are engineered through the combination of two or more biocompatible materials, play an increasingly crucial role in a wide range of clinical applications. In the field of orthopedics, nano-hydroxyapatite/polyamide 66 (n-HA/PA66) emulates the mechanical properties of natural bone and is clinically utilized in transforaminal lumbar interbody fusion (TLIF) and limb-sparing tumor reconstruction [66]. For skin repair, electrospun collagen fibers blended with chitosan or polycaprolactone (PCL) offer biomimetic, porous architectures that facilitate cell infiltration, angiogenesis, and controlled wound healing, rendering them suitable for advanced dressings and regenerative scaffolds [67]. In the area of targeted drug delivery, thermosensitive poly(N-isopropylacrylamide)/Fe_3_O_4_ magnetic microparticles enable spatiotemporally controlled release under external stimuli (e.g., alternating magnetic fields or mild hyperthermia) [68]. Cardiovascular tissue engineering benefits from chitosan-L-arginine complexes, the synergistic bioactivity of which enhances endothelial cell adhesion, nitric oxide production, and antithrombogenicity.

**Figure 5:**
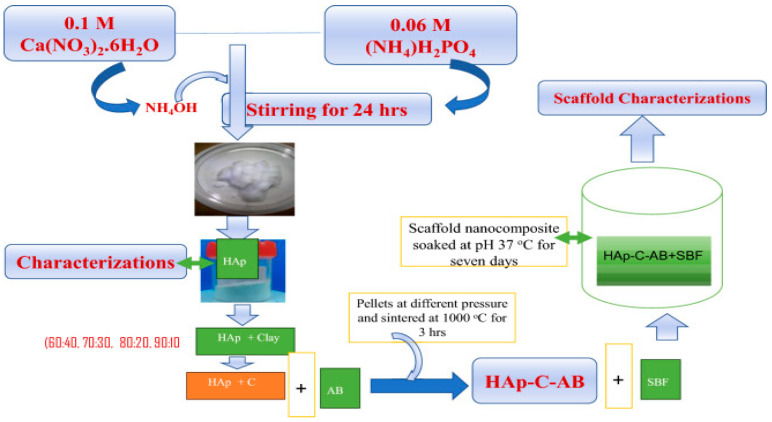
Flow chart for the production of HAp-C-AB composite and its biological applications [71].

Crucially, composite design capitalizes on component complementarity to overcome the inherent limitations of single-material systems. For instance, blending polylactic acid (PLA) with hydroxyapatite (HA) substantially improves tensile strength and fracture toughness while regulating degradation kinetics, thereby enabling sustained protein adsorption and the gradual release of bioactive factors [69]. Beyond structural reinforcement, modern medical composites possess multiple functions. Polyurethane/montmorillonite nanocomposites loaded with chlorhexidine acetate demonstrate long-lasting, contact-independent antibacterial activity [70] and are applicable in bone tissue engineering. Relevant research has developed hydroxyapatite/clay nanocomposites, which can effectively enhance mechanical properties and adjust the pH of body fluids to the physiologically suitable range. These nanocomposites exhibit favorable in vitro mineralization capacity and are well-suited for bone tissue engineering applications (Figure 5) [71].

Polylactic acid (PLA) and its composites, which are fabricated into scaffolds, films, and hydrogels through 3D printing and electrospinning, show potential for biomedical applications. Nevertheless, significant challenges impede their clinical translation: (1) The interfacial weakness between PLA and reinforcing components (such as natural fibers or nanoparticles) impairs mechanical integrity, particularly under dynamic physiological loading, as demonstrated by the premature failure in PLA/fiber composites resulting from accelerated interfacial degradation; (2) the poor interfacial controllability undermines the precise adjustment of degradation kinetics and structural performance; (3) the acidic degradation by-products of PLA can reduce the local pH, potentially leading to inflammatory responses; (4) nanoscale or metallic additives raise biosafety concerns. For instance, the agglomeration of alumina nanoparticles may cause cytotoxicity [72], and the galvanic coupling in Ti-Mg composites accelerates corrosion-driven degradation; and (5) the interspecies differences between preclinical animal models and human physiology restrict the predictive validity of in vivo performance data. Therefore, certain scholars implement multi-material compounding strategies to improve the mechanical strength and morphological stability of materials. For example, pure spider silk hydrogels exhibit satisfactory biocompatibility and high cell viability. However, they face challenges such as weak cell proliferation capacity and poor mechanical properties at low concentrations. In related studies, low-concentration spider silk hydrogels were modified through the layer-by-layer integration of electrospun spider silk fiber networks. While maintaining excellent biocompatibility, this method effectively reconciles cell proliferation ability and mechanical performance, providing a reliable reference for the development of high-performance spider silk hydrogels in the field of soft tissue engineering [73].

To overcome these limitations, next-generation medical composites are increasingly designed with functionalization (e.g., bioactive signaling), intelligence (e.g., stimuli-responsive degradation), and personalization (e.g., patient-specific geometry and degradation profiles) as core objectives.

### 2.4. Medical Inert Materials: Assurance of Long-Term Stability

Medical inert materials are biocompatible substances that maintain chemical stability and display minimal interaction with the surrounding tissues or physiological fluids. Historically, first-generation biomaterials, such as ceramics, metals, and alloys, were predominantly inert, characterized primarily by their resistance to chemical degradation and mechanical durability in the biological environment.

These materials attain biocompatibility mainly through surface passivation, such as the spontaneous formation of a stable TiO_2_ layer on titanium alloys, or intrinsic structural stability, as demonstrated by the chemical inertness and chain robustness of polypropylene. Nevertheless, their biological inertness presents a double-edged situation: while it minimizes immune activation and reduces fibrous encapsulation, it also prevents active involvement in tissue regeneration or functional integration with the host’s biological system. The key advantages are as follows: (1) Biocompatibility—chemical inertness suppresses inflammatory and allergic responses; for example, polypropylene has well-documented non-toxicity and non-allergenicity in clinical applications. (2) Mechanical performance—titanium alloys and PEEK provide high tensile strength, excellent fatigue resistance, and dimensional stability under cyclic loading [74]. (3) Corrosion resistance—metals such as 316 L stainless steel preserve their integrity in physiological environments due to protective surface oxide films [75]. (4) Economic feasibility—raw materials are plentiful, and processing methods (e.g., injection molding of medical-grade silicone rubber) allow for scalable and cost-effective production. (5) Sterilization compatibility—many materials maintain their structural integrity after autoclaving (e.g., PEEK can endure repeated 121 °C, 2-bar steam sterilization) [76]. The primary limitations of biologically inert biomaterials are as follows: (1) suboptimal osseointegration resulting from their intrinsic chemical inertness. For instance, titanium alloys need surface modifications (such as hydroxyapatite coating or micro/nano-topography) to achieve stable bone bonding. (2) Non-biodegradability, which prevents natural resorption and frequently necessitates secondary removal surgery, as is the case with conventional stainless steel or cobalt–chromium orthopedic implants. (3) Stress shielding, caused by the mismatch in elastic modulus with cortical bone (usually 10–30 GPa), which leads to peri-implant bone resorption. This is particularly prominent in rigid cobalt–chromium alloys (modulus approximately 200 GPa). (4) Imaging interference, especially with radiolucent materials like PEEK, which have low X-ray attenuation characteristics, thereby impeding the postoperative assessment of bone healing or implant positioning [77]. (5) Substandard surface biofunctionality, such as low hydrophilicity or uncontrolled protein adsorption, as seen in polymers like styrene-based elastomers [78]. This compromises cell adhesion and early tissue integration. Despite these constraints, inert biomaterials remain clinically essential [79]. In orthopedics and dentistry, titanium alloys are regarded as the gold-standard materials for joint replacements and dental implants; zirconia ceramics offer esthetic [80], high-strength solutions for crowns and abutments. In cardiovascular applications, nickel–titanium (Nitinol) stents with diamond-like carbon (DLC) coatings improve corrosion resistance and hemocompatibility [81], while braided polyester vascular grafts mimic the mechanical compliance and porosity of native arteries [82]. In soft-tissue reconstruction, silicone-based elastomers utilize their outstanding flexibility, long-term stability, and minimal immunogenicity for breast prostheses and auricular reconstructions [83].

Inert medical ceramics are typically fabricated through solid-state sintering, isostatic pressing, the sol–gel method, and photocurable 3D printing. These techniques produce dense and mechanically robust implants and bone repair components. Nevertheless, they are subject to several significant limitations, including the requirement for high-temperature sintering, non-uniform shrinkage of the green body, and residual internal porosity. Additionally, the resulting ceramics display intrinsic brittleness, low fracture toughness, and negligible bioactivity, which are key drawbacks that hinder effective osseointegration with the host bone tissue. To address these challenges, strategic surface modification has become an essential measure for enhancing their biological functionality and clinical performance.

However, several crucial challenges persist in the clinical translation of this technique. Firstly, the bioinert characteristic of current implant materials restricts their osteoinductive ability, necessitating surface modifications such as micro-arc oxidation or laser texturing to facilitate bone formation [84]. Secondly, the degradation behavior remains inadequately controllable. For degradable metals such as magnesium and zinc alloys, attaining an optimal equilibrium between mechanical integrity and predictable degradation kinetics still poses a significant obstacle. Thirdly, the long-term in vivo stability is undermined by the intricate physiological environment, encompassing variable pH, enzymatic activity, and temperature fluctuations. This can induce coating delamination (e.g., diamond-like carbon films) or excessive metal ion release (e.g., from nickel–titanium alloys), thereby raising concerns regarding biocompatibility and safety.

**Figure 6:**
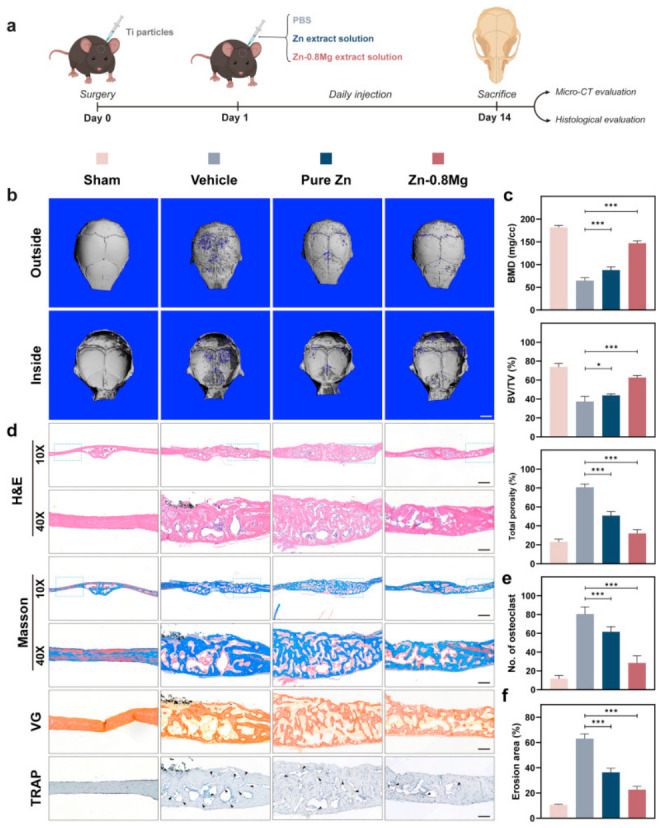
Zn-0.8 Mg alloy inhibited osteoclast activation in a mouse calvarial osteolysis model. (**a**) Experimental outline of mouse cranial osteolysis model construction. (**b**,**c**) Micro-CT images of the skull at 14 days (**b**) and quantification of bone mineral density (BMD), bone volume per total volume (BV/TV), and total porosity (**c**) in the mouse cranial bone (n = 5). Scale bar: 2 mm. (**d**,**e**) Representative H&E, Masson, Van Gieson (VG), and TRAP staining images. TRAP-positive osteoclasts are indicated by black arrows. (**e**,**f**) Quantitative analysis of osteoclast numbers and bone resorption area from TRAP immunohistochemistry staining (n = 5). Scale bar: 500 μm (10×), 200 μm (40×) [86], * *p* < 0.05, *** *p* < 0.001.

The majority of researchers enhance the performance of biomaterials via surface modification and compositional engineering. For example, coating titanium implants with strontium-doped hydroxyapatite substantially improves osteogenic activity, thereby promoting more rapid bone integration [85]. Simultaneously, degradable zinc-based alloys (e.g., Zn-Mg-Ca) have emerged as prospective alternatives in orthopedics, as their corrosion rate aligns closely with the natural bone-healing timeline (Figure 6) [86]. Moreover, porous gradient titanium alloys, such as Ti-13Nb-13Zr, reduce the elastic modulus mismatch with native bone and offer interconnected pore architectures that facilitate vascularization and new bone ingrowth [87]. Wu et al. employed the intrinsic breathing effect of MIL-53 (Fe) to enhance the interaction between titanium alloy scaffolds and vascular endothelial cells, thus improving the angiogenesis-inducing capacity of the scaffolds, particularly during sprouting angiogenesis. Following modification through these strategies, inert ceramics integrate the high strength and high stability of the substrate with the excellent bioactivity of the coatings. They are capable of not only meeting the long-term service requirements of dental restoration and artificial joints but also accelerating osseointegration and shortening the healing period after implantation [88].

Despite a multitude of pressing clinical challenges, inert biomaterials continue to be the predominant choice for medical implants, which are mainly valued for their well-established stability and favorable mechanical performance. However, their inherent biological passivity and limited bioactivity restrict therapeutic outcomes, propelling innovation towards three strategic directions: (1) dynamic surface engineering to improve host integration; (2) intelligently designed biodegradable systems that align with tissue regeneration timelines; and (3) multifunctional composites that combine structural integrity with biological signaling. Thus, the realization of next-generation implants will require profound interdisciplinary collaboration, not only between materials science and bioengineering but also across immunology, mechanobiology, and clinical translation, to co-design materials that are not only mechanically strong but also biologically informative.

### 2.5. Medical Active Materials: Actively Regulating Physiological Processes

Medical active materials are a category of advanced biomaterials that are engineered to engage in dynamic interactions with biological systems. They do not merely coexist with tissues; rather, they actively direct physiological responses. In contrast to passive biocompatible materials, these active materials respond to local biochemical or physical signals (such as pH, enzymes, and mechanical stress) to initiate precise functions. These functions include stimulating bone regeneration through osteoinductive surface topographies, releasing therapeutic agents in a spatiotemporally regulated manner, or degrading into pro-angiogenic ions like Mg^2+^. The design of medical active materials incorporates three fundamental requirements. Firstly, there is basic biocompatibility, which is characterized by non-toxicity, the absence of chronic inflammation, and immune rejection. Secondly, programmable bioactivity is achieved via customized surface chemistry, nanostructured interfaces, or stimuli-responsive compositions. Thirdly, functional accountability demands that each feature of the material be rationally associated with a measurable biological outcome. The crystallographic alignment of hydroxyapatite and the controlled corrosion kinetics of magnesium alloy serve as examples of how deliberate structural and compositional engineering can lead to clinically relevant biological effects.

This category of biomaterials predominantly consists of hydroxyapatite (HA), β-tricalcium phosphate (β-TCP), biphasic calcium phosphate (BCP), calcium silicate, and bioactive glass. Structurally and compositionally similar to the inorganic phase of natural bone, these materials display remarkable biocompatibility, osteoconductivity, and inherent osteogenic potential. Their high compressive strength and elastic modulus allow for the fabrication of porous 3D scaffolds, bioactive coatings, and injectable or granular fillers for bone defect reconstruction. Nevertheless, their intrinsic brittleness and low fracture toughness restrict their use in load-bearing applications. To address this limitation, they are typically hybridized with natural polymers, such as silk fibroin, spider silk protein, or chitin derivatives, to synergistically enhance mechanical resilience, facilitate cell adhesion and proliferation, and more effectively mimic the native extracellular matrix. A notable example is the β-TCP aerogel functionalized with a polyvinyl alcohol/chitin fiber network (BTCP-AE-FMs), developed by Boda et al. This composite not only expedited mineralized tissue formation in vitro but also significantly increased new bone volume and bridging in critical-sized calvarial defects in vivo, demonstrating strong translational potential as a next-generation bone substitute [89].

Bioactive glass, an inorganic biomaterial, is well-known for its remarkable bioactivity and outstanding biocompatibility. Once implanted, it quickly integrates with the native bone tissue, achieving true osseointegration, and concurrently exerts osteoconductive and osteogenic effects. In addition to providing structural support, it actively regulates cellular behavior (e.g., promoting the proliferation and differentiation of osteoblasts) and alleviates local oxidative stress. Its adjustable particle size allows for the easy fabrication of uniform microspheres, which are suitable for the high-efficiency loading and controlled release of therapeutic agents such as quercetin. Particularly adapted to the moist, dynamic, and microbiologically complex environment of periodontal tissues, bioactive glass is increasingly combined with hydrogels, especially photocurable and injectable formulations, to create localized, sustained-release delivery systems. These hybrid systems not only stably retain drugs at the defect site but also synergistically enhance the osteogenic differentiation of stem cells, rendering them highly promising for the regeneration of periodontal bone defects. For instance, Zhu et al. developed a photocurable, injectable hydrogel embedded with nano-sized bioactive glass microspheres. This system successfully delivered quercetin in situ, maintained the therapeutic concentration under physiological conditions, and significantly accelerated osteogenic regeneration in preclinical models of periodontal bone loss [90].

The surface chemical properties of bioactive materials, such as hydroxyapatite and bioactive glass, play a crucial role in initiating cellular responses. They facilitate cell adhesion and proliferation, establish direct chemical bonds with the host bone tissue, and substantially reduce foreign-body reactions. For instance, bone-regenerative scaffolds imitate the mineral composition of natural bone, thus enhancing osteoblast differentiation and expediting defect healing. Biodegradable polymers, such as polylactic acid (PLA), degrade in vivo into non-toxic metabolites (e.g., lactic acid), obviating the necessity for surgical removal [91]. Propelled by bionic design and multifunctional integration, these materials are propelling the advancement of precision regenerative medicine. However, several critical challenges persist: (1) the long-term stability of materials under physiological conditions; (2) scalable and reproducible manufacturing, particularly for high-purity nanoceramics, which demand strict synthesis protocols and expensive infrastructure; (3) comprehensive biosafety assessment, encompassing the risk of chronic inflammation, the metabolic burden caused by acidic degradation byproducts (e.g., the local pH drop induced by PLA impairs cell viability), and the potential bioaccumulation of nanomaterials; and (4) translational fidelity. Many angiogenic or osteoinductive materials demonstrate strong efficacy in murine models but fail to reproduce their function in human clinical settings because of inter-species differences in immune response, vascular complexity, and tissue microenvironment.

## 3. Status and Challenges of Biomedical Materials

### 3.1. Current Development Status: Synergy Among Policy, Technology

Policy support: The People’s Republic of China has placed a high priority on the development of biomedical materials and actively promoted their alignment with international standards. In this regard, regulatory authorities have introduced specific incentive policies, including direct research and development (R&D) funding, tax incentives for innovation-driven enterprises, and a dedicated “fast-track” approval pathway for novel biomedical materials. Significantly, China’s increased involvement in international standard-setting bodies, such as ISO/TC 10993-12 [92] and ASTM F04 [93], not only enhances the global recognition of domestic innovations but also improves market access, competitiveness, and cross-border collaboration for Chinese manufacturers. This comprehensive policy framework provides a solid foundation for the sustainable development of the biomedical materials sector.

**Figure 7:**
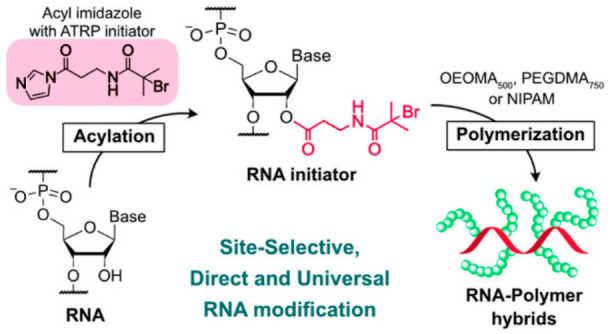
Manufacturing of RNA-synthesized polymer hybrid materials [96].

Technological breakthroughs in biomedical engineering. Recent advancements in biomedical engineering have led to the development of several transformative technologies with substantial clinical potential. Firstly, 3D bioprinting facilitates the production of patient-specific tissue scaffolds and programmable drug-delivery systems. In particular, it can create spatially precise spinal cord injury repair scaffolds that replicate the native tissue architecture [94]. Secondly, femtosecond laser surface modification enables micronano-scale topographic patterning on metallic implants. This not only enhances osseointegration but also achieves an antibacterial efficacy of over 99% against common pathogens such as *S. aureus* and *E. coli*. Thirdly, pH-sensitive hollow mesoporous carbon nanospheres (HMCNs) promote tumor-targeted drug release through a calcium-gated mechanism. They take advantage of the acidic microenvironment of solid tumors to trigger the release of the payload [95]. Finally, RNA-polymer hybrid materials have been synthesized using a dual-controlled strategy: site-selective acylation of ATRP initiators followed by visible-light-induced polymerization (Figure 7) [96]. This strategy allows for unparalleled control over RNA stability, cellular delivery, and spatiotemporal activity.

New materials for biomedical applications: Polybutylene succinate (PBS) functionalized with aspartic acid side chains significantly enhances cell adhesion and facilitates precise drug targeting [97]. Polymer-coated Fe_3_O_4_ magnetic nanoparticles combine high-contrast T2-weighted magnetic resonance imaging (MRI) with efficient non-viral gene delivery [98]. Moreover, niobium-and zirconium-doped high-entropy titanium alloys display an elastic modulus of 30–50 GPa, which is well-matched to human cortical bone, thus reducing stress shielding and enhancing long-term implant osseointegration [99].

Market demand: The global biomedical materials market is witnessing sustained growth, primarily propelled by three interrelated trends: demographic aging, increasing health awareness, and infrastructure development in emerging economies. Firstly, the aging population, as exemplified by China, where 15.38% of the population is aged 60 or above, is significantly increasing the incidence of age-related diseases such as osteoarthritis and cardiovascular disease. This, in turn, is accelerating the demand for artificial joints, coronary stents, and bone-and vascular-regenerative materials. Secondly, higher living standards and greater public awareness of preventive and personalized healthcare are raising the requirements for the safety, efficacy, and accessibility of medical devices. This is driving innovation towards advanced, biocompatible, and functionally customized materials. Thirdly, rapid urbanization and economic development in Asia, Africa, and Latin America are expanding hospital capacity and modernizing clinical infrastructure, generating strong demand for both basic and next-generation biomedical products, ranging from antimicrobial hydrogel dressings and absorbable sutures to ceramic–polyethylene acetabular components [100]. Collectively, these factors identify chronic wound management, minimally invasive implantation, and regenerative orthopedics as the key growth areas of the sector.

### 3.2. Core Challenges: The Key Bottlenecks Restricting the Industry’s Development

#### 3.2.1. Security Risks

The safety of medical biomaterials serves as the cornerstone for their clinical translation and practical application in the real-world, encompassing every phase of their life cycle, from the design and manufacturing stages to pre-clinical testing and in vivo utilization. Ensuring safety necessitates a comprehensive, risk-based assessment of five mutually dependent aspects: biocompatibility, chemical stability, mechanical performance, degradation behavior, and long-term biological response. Among these factors, biocompatibility holds the utmost importance. Materials should not induce cytotoxicity, immune activation, or hemocompatibility problems when in contact with human tissues or physiological fluids. For instance, as specified by ISO 10993, implantable devices are subjected to a tiered biological evaluation, which includes tests for cell viability, cytokine release, and hemolysis. Moreover, cardiovascular biomaterials are required to exhibit minimal platelet adhesion and thrombogenic potential. Similarly, orthopedic implants need to possess mechanical properties that closely align with those of native bone to prevent stress shielding and subsequent bone resorption [101].

Toxicological evaluation centers on the intrinsic hazards of biomaterials and their degradation by-products, such as metal ions or polymer monomers. It is particularly crucial for degradable implants, like polylactic acid (PLA) and magnesium alloys. Comprehensive testing should encompass assessments of acute and chronic toxicity, teratogenicity, and carcinogenicity. Significantly, the degradation kinetics should be in line with the rate of tissue regeneration, and all metabolites must be biocompatible and easily eliminated. For example, lactic acid from PLA is completely metabolized via the tricarboxylic acid cycle into carbon dioxide and water, imposing no systemic burden [102].

Ensuring safety throughout the medical device manufacturing process is of critical importance. This necessitates strict control over the purity of raw materials, precise optimization of sterilization methods, which encompass steam autoclaving, gamma irradiation, and ethylene oxide gas, as well as careful selection and validation of surface modification techniques. Residual monomers, catalysts, or additives (e.g., plasticizers) present potential toxicity risks. Similarly, aggressive sterilization conditions may lead to the degradation of polymers or the generation of harmful leachables, such as aldehydes from ethylene oxide residues or free radicals from irradiation. For surface-modified implants, such as hydroxyapatite-coated titanium alloys, the adhesion, uniformity, and long-term stability of the coating must be rigorously verified to prevent delamination, which may trigger local inflammation or systemic immune responses. Preclinical evaluation adopts a tiered testing approach, including cytocompatibility assays (ISO 10993-5 [103]), hemocompatibility studies (ISO 10993-4 [104]), and well-designed animal models to assess acute and chronic effects, such as fibrosis, granuloma formation, and delayed hypersensitivity. Clinical trials then build upon this foundation through phased, ethics-approved human studies that prospectively monitor implant-related infections, immune rejection, functional integration, and rare but serious long-term outcomes, such as carcinogenicity or aberrant tissue encapsulation. Long-term safety encounters complex challenges. The material may undergo performance degradation in the vivo environment because of mechanical fatigue (e.g., heart valves), wear (e.g., artificial joints), or corrosion (e.g., the release of cobalt and chromium ions from metal implants), which increases the risk of secondary surgery. If the degradation rate of biodegradable materials does not match tissue repair, it may lead to structural collapse or inflammatory responses. The long-term retention of non-biodegradable materials, like silicone prostheses, may trigger chronic inflammation or fibrotic wrapping [105].

Moreover, material aging is influenced by both physiological conditions, such as pH levels and enzymatic activity in bodily fluids, and external environmental factors, including ultraviolet radiation and temperature fluctuations. Therefore, the prediction of the material’s service life should be incorporated into the early design phase.

#### 3.2.2. The Prohibitively High Cost of Biomedical Materials

The development and manufacturing of biomedical materials entail a highly intricate, time-consuming, and capital-intensive process. At the research and development stage, a significant amount of investment is necessary, not only for fundamental research but also for rigorous pre-clinical testing, regulatory submissions, and multi-phase clinical trials. For instance, introducing a novel biodegradable biomaterial to the market typically takes 8–12 years and incurs costs exceeding $100 million, mainly because of extensive safety validation, biocompatibility assessments, and efficacy confirmation in human trials. During the manufacturing process, compliance with ISO 13485 [106] and FDA cGMP standards requires specialized facilities, strict raw material traceability, validated sterilization protocols, and continuous equipment calibration, which are factors that substantially increase production costs. These financial burdens restrict industry profitability, raise end-user prices, and ultimately curtail patient access and broader clinical adoption of life-enhancing biomaterials.

Moreover, cost continues to be a critical challenge, which is closely linked to production scale and supply chain efficiency. Biomedical materials cater to a highly specialized and relatively small market, which makes it challenging to achieve economies of scale. Additionally, their supply chains are inherently complex, and the strict quality requirements for raw materials increase procurement and processing costs. Consequently, reducing costs without sacrificing safety, efficacy, or regulatory compliance is one of the most urgent industry-wide challenges at present.

#### 3.2.3. Lagging Regulation and Insufficient Coordination

Biomedical materials have a direct impact on human health, necessitating strict regulatory supervision. However, the rapid progress of technology and the subsequent influx of novel materials and therapies are putting pressure on the existing regulatory frameworks. Firstly, regulatory standards frequently fall behind innovation, resulting in crucial gaps in the safety and efficacy evaluation of advanced biomaterials. Secondly, the fragmented regulatory policies among different countries pose significant obstacles to global product registration and market access, complicating international harmonization and delaying patients’ access to life-saving innovations.

#### 3.2.4. Inadequate Maintenance System

Regular maintenance is of utmost importance for biomedical materials, particularly implantable devices like pacemakers, which are intended for long-term use within the body. These devices necessitate periodic clinical assessment, calibration, and software updates to guarantee safety, reliability, and therapeutic effectiveness. However, the current maintenance infrastructure remains fragmented. There is a shortage of specialized technicians, geographical access is uneven, service coverage is restricted, and out-of-pocket expenses can be extremely high. Equally worrying are the persistent deficiencies in data security and patient privacy protection during remote monitoring, firmware updates, or device retrieval. In the absence of coordinated policy actions, standardized protocols, and equitable service expansion, these systemic drawbacks pose a risk of undermining clinical outcomes, eroding patient trust, and jeopardizing long-term treatment success.

#### 3.2.5. Immune Rejection Issues Associated with Biomedical Materials

Despite substantial advancements in the biocompatibility of biomedical materials, immune-mediated rejection persists as a chronic and clinically significant challenge. The human immune system is highly sensitive to detect molecular “non-self” signals. To such an extent, even highly refined synthetic or bioengineered implants can incite strong inflammatory and adaptive immune responses. For instance, in organ transplantation, rejection takes place across all graft types, even when using HLA-matched donors and the most advanced immunosuppressive regimens, which results in graft dysfunction, fibrosis, or failure. This immune activation not only impairs implant integration and long-term functionality but also endangers patient survival. Consequently, lifelong immunosuppression is frequently necessary. However, this approach entails considerable risks, such as opportunistic infections, metabolic disorders, and heightened susceptibility to malignancy, ultimately increasing morbidity, mortality, and the healthcare burden.

To surmount the enduring challenge of immune rejection, researchers are implementing three complementary strategies: (1) engineering “stealth” biomaterials that avoid immune detection by modulating surface properties, such as nanoscale roughness, a controlled balance of hydrophilicity and hydrophobicity, and customized functional groups; (2) optimizing material chemistry, for example, adjusting the cross-linking density of polymers or incorporating immunomodulatory moieties; and (3) integrating materials with biological interventions, including the localized delivery of regulatory cytokines, patient-specific T-reg cell therapy, and CRISPR-based editing of immune checkpoint pathways. Collectively, these integrated approaches are intended not only to suppress acute and chronic rejection but also to promote long-term biointegration, thereby ultimately enhancing implant survival, clinical efficacy, and the quality of life of patients.

#### 3.2.6. Examples of Special Risks: Failure Modes of Artificial Heart Valves

Artificial heart valves present a fundamental clinical dilemma: no existing design can balance long-term durability with physiological compatibility. Mechanical valves, which are manufactured from pyrolytic carbon and titanium alloys, provide remarkable structural longevity. However, they demand lifelong anticoagulation, exposing patients to annual risks of thromboembolism or major bleeding (2–8%) and subclinical hemolysis due to repetitive leaflet impact. In contrast, bioprosthetic valves, sourced from decellularized porcine or bovine pericardium, imitate native hemodynamics and eliminate the need for chronic anticoagulation. Nevertheless, they undergo progressive structural degeneration, primarily through calcification, which necessitates reoperation in approximately 30% of recipients within 10–15 years. Significantly, these biological limitations are not merely mechanical. Residual xenogeneic antigens, even after thorough decellularization, initiate low-grade immune activation that actively speeds up calcification and tissue failure. This biological factor exacerbates the economic burden. Although device costs range from ¥10,000 to ¥50,000, lifetime expenses, including INR monitoring, dietary counseling, serial echocardiography, warfarin–vitamin K interaction management, and potential reintervention, often exceed the initial implantation costs by several times. Consequently, both valve types require distinct, lifelong surveillance models. Recipients of mechanical valves need strict anticoagulation control and careful monitoring of drug-diet interactions, while bioprosthetic valve recipients depend on early imaging-based detection of functional decline. Ultimately, valve failure almost always requires rethoracotomy, a procedure with significantly higher morbidity, mortality, and technical risk than primary implantation. This emphasizes that the choice between valve types is not merely a technical issue but a continuous negotiation among clinical safety, biological fidelity, and long-term resource allocation.

Patients who have mechanical heart valves are in need of lifelong anticoagulation management, which encompasses regular INR monitoring and a vigilant approach to drug–food interactions, especially foods rich in vitamin K, as these can notably diminish the anticoagulant effect of warfarin. Conversely, patients with bioprosthetic (biological) valves generally do not necessitate long-term anticoagulation; however, they still need periodic echocardiographic or cardiac MRI follow-ups to evaluate the structural integrity and hemodynamic function. Significantly, both groups are required to adhere to a structured daily self-monitoring regime and report symptoms promptly, as the delayed recognition of valve degeneration, thrombosis, or paravalvular leak can potentially lead to acute heart failure or embolic events. When valve failure takes place, surgical reintervention, most commonly re-sternotomy for valve replacement, is associated with a substantially higher morbidity and mortality rate compared to the initial implantation. This is attributed to the increased technical complexity, poorer myocardial tolerance, greater bleeding risk, and higher susceptibility to postoperative complications. As a result, secondary surgery imposes more stringent preoperative physiological requirements, extends the recovery period, and significantly increases both the clinical risk and the socioeconomic burden. From a regulatory perspective, comprehensive post-market surveillance, including mandatory adverse event reporting, real-world performance registries, and proactive signal detection, is crucial for the continuous evaluation of the safety and durability of prosthetic heart valves [107].

Typical failure cases of artificial heart valves underscore critical challenges throughout the device lifecycle. In April 2025, during the implantation of the Medtronic Regent mechanical heart valve, an oversized prosthesis led to incomplete leaflet opening. Despite an emergent valve replacement, the patient died due to a hemorrhagic rupture at the aortic suture site, which was subsequently documented by the FDA in its death-related report (Case No. 2135147-2025-02495). Similarly, the Portico transcatheter aortic valve was recalled after clinical evidence associated stent frame deformation with early thrombosis. Moreover, Rizwan et al. indicated that glutaraldehyde-fixed bioprosthetic valves, despite their widespread use, are susceptible to progressive calcification and structural deterioration, mainly caused by xenograft-mediated immune responses. Collectively, these real-world incidents, sourced from clinical practice and regulatory databases, reveal persistent, system-level risks covering design validation, surgical technique, and long-term hemodynamic compatibility. They highlight a fundamental shortcoming: current biomedical materials still lack the durability, bioinertness, and immune compatibility necessary for truly safe, lifelong cardiovascular device implantation.

## 4. Future Trends in Biomedical Materials

### 4.1. CIntelligence and Functional Precision

Medical biomaterials are on the verge of becoming a driving force, rather than merely a supporting component, in next-generation healthcare. The future is centered around intelligent and adaptive materials, which are systems capable of sensing physiological cues, responding in real-time, and evolving in tandem with the body. Facilitated by the synergistic progress in nanotechnology, biosensors, and AI-driven design, these next-generation biomaterials surpass traditional biocompatibility and mechanical functionality. They actively regulate drug release in response to fluctuations in local pH, temperature, or biomarkers; they adjust their stiffness or degradation rate to align with tissue healing stages; and, powered by 4D printing, they undergo transformation post-implantation, unfolding, contracting, or self-assembling to meet the dynamic anatomical and functional requirements. This transition from passive scaffolds to responsive, life-integrated therapeutics represents a paradigm shift in precision medicine [108]. For example, Chen et al. developed a glucose-responsive intelligent antioxidant hydrogel leveraging reversible boronic acid-diol interactions. Under hyperglycemic conditions, the hydrogel undergoes structural modulation to enable on-demand, sustained release of therapeutic antioxidants. It exhibits excellent biocompatibility, tunable biodegradability, and robust yet elastic mechanical properties suitable for dynamic wound sites. Functionally, it efficiently scavenges reactive oxygen species (ROS), suppresses pro-inflammatory cytokine expression, promotes endothelial cell migration and tubule formation (angiogenesis), and enhances type I/III collagen deposition and alignment—collectively accelerating the closure and functional restoration of chronic diabetic wounds. This work establishes a rationally designed, stimuli-responsive platform that bridges smart biomaterials with pathophysiology-informed wound healing [109].

### 4.2. Renewable and Eco-Friendly

Concurrently, research on renewable and biodegradable biomaterials is on the verge of rapid progress. Propelled by the global trend towards sustainable development, the design of next-generation medical biomaterials increasingly emphasizes environmental compatibility, resource efficiency, and clinical safety. Natural polymers, such as collagen, chitosan, and hyaluronic acid, along with synthetic biodegradable substitutes like polylactic acid (PLA), polyglycolic acid (PGA), and their copolymers (e.g., PLGA), are emerging as prominent candidates. Significantly, these materials not only degrade predictably in vivo, thus minimizing the necessity for removal surgeries, but also leave a minimal ecological footprint after disposal. Moreover, the synergies with tissue engineering and regenerative medicine are expediting the clinical translation of bioactive platforms, especially stem-cell-laden scaffolds engineered to direct functional tissue regeneration [110].

### 4.3. Personalization and Precision Medicine Fit

Personalized and precise medical care is propelling a revolution in medical biomaterials, thereby initiating an era of genuinely patient-specific solutions. Progressions in genomics, proteomics, and bioinformatics currently enable clinicians and material scientists to customize biomaterials according to individual patients’ genetic profiles, disease subtypes, and real-time physiological conditions. Simultaneously, high-resolution 3D bioprinting has advanced from prototyping to facilitate the clinical production of anatomically precise, functionally graded implants, such as load-bearing joint replacements, bioactive dental scaffolds, and endothelialized vascular stents, which significantly improves therapeutic efficacy and long-term patient outcomes. Complementing these developments, AI-powered material design platforms, trained on multimodal biomedical datasets, are expediting discovery, forecasting structure–property–function relationships, and shortening the translational timeline from the laboratory to the patient’s bedside.

### 4.4. Interdisciplinary Innovation

Interdisciplinary integration and technological innovation are on the verge of revolutionizing the field of medical biomaterials. Through the convergence of materials science, biology, medicine, engineering, and information technology, researchers are leading the way in three transformative frontiers: (1) Bionic biomaterials, which are engineered to replicate the structure and function of native tissues for seamless bio-integration; (2) bioelectronic materials, which enable intelligent, implantable devices such as closed-loop neural interfaces and adaptive cardiac pacemakers; and (3) CRISPR-integrated biomaterials, which are spatially and temporally controlled delivery platforms that precisely modulate cell behavior to unlock next-generation regenerative and immunomodulatory therapies [111].

## 5. Conclusions and Perspectives

### 5.1. Conclusions

Biomedical materials have undergone an evolution from passive structural replacements to dynamic, functionally integrated agents that actively engage in biological processes, such as modulating immune responses, guiding tissue regeneration, and facilitating spatiotemporally controlled therapeutic delivery. This review systematically assesses the recent advancements across four crucial material classes: metallic systems (e.g., bioresorbable Mg alloys), synthetic and natural polymers (including stimuli-responsive hydrogels), hybrid composites (e.g., polymer-ceramic scaffolds), and smart inert/active platforms (such as nitric oxide-releasing coatings or enzyme-mimetic nanomaterials) (Table 1). We emphasize how each category tackles critical clinical challenges in orthopedics, cardiovascular repair, targeted drug delivery, and regenerative medicine. These innovations are being accelerated by a powerful triad: evidence-based regulatory pathways, cross-disciplinary technological integration (e.g., AI-driven material design and 3D bioprinting), and the increasing global demand, particularly propelled by aging populations and the shift towards preventive, personalized healthcare.

However, the translation of biomedical materials from laboratory settings to widespread clinical application continues to be confronted with substantial challenges. Firstly, safety and biocompatibility necessitate rigorous and long-term assessment, encompassing not only acute toxicity and immune reactions but also degradation kinetics, metabolite profiles, and tissue-level integration over an extended period. Secondly, high development costs, which cover research and development, Good Manufacturing Practice (GMP)-compliant manufacturing, and strict quality assurance, restrict scalability and equitable access. Thirdly, regulatory frameworks are lagging behind scientific progress. Fragmented national guidelines impede global trials and delay approvals, highlighting an urgent requirement for harmonized, science-based international standards. Finally, persistent technical obstacles include host immune rejection, implant instability (such as stress shielding, particle-induced osteolysis, or interfacial fibrosis), and the absence of real-time, non-invasive monitoring tools for post-implantation performance and maintenance. Despite these complications, the field is making decisive progress towards true biological integration, where next-generation biomaterials do not merely coexist with living systems but interact dynamically, adapt to, and even guide them. The convergence of material science, synthetic biology, mechanobiology, and AI-driven analytics is redefining the frontier of regenerative medicine.

### 5.2. Future Perspectives

Biomedical materials are on the verge of experiencing a revolutionary transformation, propelled by three converging trends that jointly tackle the existing clinical limitations and unleash unparalleled therapeutic potential:

(1) Intelligent, stimuli-responsive systems: The next-generation biomaterials will incorporate biosensing components and intelligent polymers, which can enable the real-time detection of physiological indicators (such as pH, enzyme activity, or mechanical strain) and trigger precise, on-demand responses. These responses encompass programmable drug release, self-adjusting stiffness during tissue remodeling, and continuous, non-invasive monitoring of the healing process, thus introducing truly adaptive implants and intelligent wound dressings.

(2) Advanced manufacturing and patient-specific design: Well-developed 4D bioprinting, when combined with AI-powered generative design and the integration of multimodal imaging, will facilitate the fabrication of anatomically precise, functionally graded scaffolds. These scaffolds will possess spatially resolved biochemical signals (e.g., immobilized growth factors), customized mechanical zonation (e.g., mimicking the stiff cortex and soft marrow), and vascularizable microarchitectures, which will significantly enhance host integration and functional recovery in craniofacial reconstruction, cartilage regeneration, and partial organ replacement.

(3) Engineered bioactivity and synergistic multifunctionality: Instead of being passive substrates, future biomaterials will function as dynamic biological regulators. This involves rationally designed biomimetic interfaces that can spatiotemporally guide stem cell differentiation; multifunctional composites that co-deliver osteoinductive ions, broad-spectrum antimicrobial peptides, and pro-angiogenic VEGF mimetics; and hybrids compatible with neural or cardiac tissue that can support electrophysiological coupling while resisting fibrotic encapsulation.

(4) Sustainable and environmentally-friendly solutions: The impetus towards eco-conscious healthcare is expediting the development of high-performance biomaterials, specifically natural polymer-based systems (e.g., chitosan, bacterial cellulose) and fully biodegradable metallic alloys (e.g., Zn-and Fe-based). Significantly, life-cycle assessment and green manufacturing principles are no longer supplementary elements but are essential components of material design from the very beginning.

(5) In-depth interdisciplinary integration: Groundbreaking innovations will increasingly surface at the crossroads of biomedical materials with genetic engineering (e.g., CRISPR-responsive scaffolds), next-generation immunotherapies, and bioelectronic interfaces. This synergy is already facilitating the creation of implantable devices capable of dynamically regulating immune activity, achieving spatiotemporally controlled gene delivery, or restoring functionality in complex neural circuits.

In summary, biomedical materials are evolving from passive implants to intelligent, adaptive, and regenerative platforms. By addressing current scientific, translational, and regulatory challenges through continuous, team-based interdisciplinary cooperation, these materials are ready to support next-generation diagnostics, curative therapies, and ultimately, a significant extension of human healthspan.

## Figures and Tables

**Table 1:** Classification and application overview of different types of biomedical materials.

Material	Common Types	Clinical Application	Clinical Research
Medical metal materials	Stainless steel, titanium and titanium alloys, cobalt-based alloys, nickel–titanium alloys (shape memory alloys), magnesium alloys, tantalum and zirconium alloys, etc.	Artificial joints, dental implants, cardiovascular stents, heart valves, bone plates, bone screws, etc.	Xu et al. treated 18 cases of carpal collapse via four-corner arthrodesis with nickel-titanium memory alloy concentrator, achieving rapid bone fusion, no obvious complications, and marked improvements in wrist strength, mobility and pain [112].
Medical polymer materials	Hydrogel materials, nanoparticles, chitosan, collagen, sodium alginate, silicone rubber, etc.	Hemostatic materials, absorbable sutures, cartilage repair scaffolds, enamel-like polymer, brain–computer interfaces, syringes, artificial blood vessels, etc.	Yu et al. compared domestic hydrogel vascular closure device with imported ExoSeal for hemostasis after femoral artery intervention. The hydrogel device has equivalent efficacy, faster hemostasis speed and less blood loss with consistent safety, which can be preferentially applied for rapid and safe hemostasis after transfemoral intervention [113].
Medical composite materials	Chitosan/graphene oxide (CS/GO) composites, Nano-hydroxyapatite/polyamide (n-HA/PA66) composites, Collagen-based electrospun fibers combined with chitosan or polycaprolactone composites, etc.	Wound dressings, targeted drugs, limb tumor repair, skin regeneration, cardiovascular stents, antibacterial, 3D-printed bone scaffolds, composite microspheres, etc.	Zhao et al. verified that n-HA/PA66 artificial lamina has good biological stability. It can effectively inhibit epidural scar hyperplasia, avoid compression on nerves and dural sac, sustain favorable spinal canal morphology and spinal function, and serve as an effective method to prevent epidural adhesion following laminectomy [114].
Medical inert materials	Ceramic materials, Stainless steel materials, Polyester, Zirconia ceramics, Biodegradable zinc alloys, Porous titanium alloy-based gradient materials, etc.	Artificial joints, dental implants, artificial breasts, auricular prostheses, etc.	Schlichting et al. found that CAD-CAM ceramic and composite resin ultrathin occlusal veneers had similar clinical effects in repairing worn posterior teeth. Ceramic veneers showed better stability, while composite resin ones were prone to chipping and surface wear [115].
Medical active materials	Hydroxyapatite, bioactive glass, polylactic acid, natural silk protein, spider silk protein, β-tricalcium phosphate, polycaprolactone, etc.	Bone repair, bone defect filling, artificial joint coatings, spinal fusion devices, antibacterial dressings, absorbable sutures, cartilage, nerve scaffolds, drug delivery carriers, etc.	Epstein’s study demonstrated that β-TCP combined with lamina autograft yields favorable lumbar fusion. Although single-segment fusion occurs earlier, single- and two-segment fusion achieve comparable fusion rates and clinical outcomes at 1-year follow-up [116].

## Data Availability

Not applicable.
